# Induction of p16^INK4a^ Is the Major Barrier to Proliferation when Epstein-Barr Virus (EBV) Transforms Primary B Cells into Lymphoblastoid Cell Lines

**DOI:** 10.1371/journal.ppat.1003187

**Published:** 2013-02-21

**Authors:** Lenka Skalska, Robert E. White, Gillian A. Parker, Alison J. Sinclair, Kostas Paschos, Martin J. Allday

**Affiliations:** 1 Section of Virology, Department of Medicine, Imperial College London, St Mary's Campus, London, United Kingdom; 2 Department of Biochemistry, University of Sussex, Brighton, United Kingdom; Wistar Institute, United States of America

## Abstract

To explore the role of p16^INK4a^ as an intrinsic barrier to B cell transformation by EBV, we transformed primary B cells from an individual homozygous for a deletion in the *CDKN2A* locus encoding p16^INK4a^ and p14^ARF^. Using recombinant EBV-BAC viruses expressing conditional EBNA3C (3CHT), we developed a system that allows inactivation of EBNA3C in lymphoblastoid cell lines (LCLs) lacking active p16^INK4a^ protein but expressing a functional 14^ARF^-fusion protein (p14/p16). The *INK4a* locus is epigenetically repressed by EBNA3C – in cooperation with EBNA3A – despite the absence of functional p16^INK4a^. Although inactivation of EBNA3C in LCLs from normal B cells leads to an increase in p16^INK4a^ and growth arrest, EBNA3C inactivation in the p16^INK4a^-null LCLs has no impact on the rate of proliferation, establishing that the repression of *INK4a* is a major function of EBNA3C in EBV-driven LCL proliferation. This conditional LCL system allowed us to use microarray analysis to identify and confirm genes regulated specifically by EBNA3C, independently of proliferation changes modulated by the p16^INK4a^-Rb-E2F axis. Infections of normal primary B cells with recombinant EBV-BAC virus from which EBNA3C is deleted or with 3CHT EBV in the absence of activating ligand 4-hydroxytamoxifen, revealed that EBNA3C is necessary to overcome an EBV-driven increase in p16^INK4a^ expression and concomitant block to proliferation 2–4 weeks post-infection. If cells are p16^INK4a^-null, functional EBNA3C is dispensable for the outgrowth of LCLs.

## Author Summary

Epstein-Barr virus (EBV) is a causative agent of several types of B cell lymphoma. In human B cells, EBV reduces protein levels of at least two tumour suppressors that would otherwise be activated in response to over-expressed oncogenes. These proteins are BIM, which induces cell death and p16^INK4a^, which prevents cell proliferation. Repression of both is via epigenetic methylation of histones and is dependent on expression of both EBNA3A and EBNA3C – two EBV proteins required for the transformation of normal B cells into lymphoblastoid cell lines (LCLs). In this report we have used EBV with a conditionally active EBNA3C – active only in the presence of 4-hydroxytamoxifen – together with B cells from an individual carrying a homozygous deletion of p16^INK4a^ to confirm that regulation of p16^INK4a^ expression is a major function of EBNA3C and demonstrate that if B cells lack p16^INK4a^, then EBNA3C is no longer required for EBV-driven proliferation of LCLs. Furthermore we show that early after the infection of normal B cells, EBV induces p16^INK4a^ accumulation that – if unchecked by EBNA3C (and EBNA3A) – prevents LCL outgrowth. Formal proof that p16^INK4a^ is the main target of EBNA3C comes with the production of p16-null LCLs that have never expressed functional EBNA3C.

## Introduction

The *CDKN2A* locus at human chromosome 9p21 encodes two important tumour suppressors, p16^INK4a^ and p14^ARF^ (equivalent to p19^ARF^ in mice); these proteins are both critical regulators of cell proliferation. The cyclin-dependent kinase (CDK) inhibitor p16^INK4a^ acts upstream of the cyclin D-dependent kinases (CDK4 and CDK6) and governs their phosphorylation of the retinoblastoma protein (Rb). By binding CDKs and blocking Rb phosphorylation, increased p16^INK4a^ expression leads to a G1 cell cycle arrest (reviewed in [1], [2]). In contrast, the p14^ARF^ and 19^ARF^ proteins regulate p53 stability via inactivation of MDM2, the p53-degrading ubiquitin ligase. Stabilization and activation of p53 leads to G1 and G2 arrest by inducing the CDK regulator p21^WAF1^ or apoptosis by inducing pro-apoptotic factors such as BAX ([1], [2]).

The products of *CDKN2A* are responsible for senescence or apoptosis in cells receiving unscheduled proliferative signals from mutant or deregulated oncogenes (this is sometimes called ‘oncogenic stress’) [3], [4]. As a consequence p16^INK4a^ and p14^ARF^/19^ARF^ can potentially act as barriers to immortalization of cells placed in culture and the development of cancers *in vivo*. Both genes are progressively up-regulated with tissue aging and they probably contribute to the process of aging *in vivo* by reducing reservoirs of self-renewing stem cells [3], [4]. It is now generally accepted p19^ARF^ plays a dominant role in these processes in mice, whereas p16^INK4a^ is the dominant player in human cells. Unsurprisingly, in a wide variety of human cancers *INK4a* is inactivated by gene deletion, mutation or promoter DNA methylation [1], [3]. The *CDKN2A* locus is regulated epigenetically by polycomb complex-generated histone modifications [1], [5] and recently it has been demonstrated that the products of the locus act as a major barrier to the reprogramming of differentiated cells into induced pluripotent stem cells. As in the other biological contexts described above, p16^INK4a^ dominates over p14^ARF^ as the critical polycomb-regulated barrier to de-differentiation in human cells [6].

Epigenetic (ie heritable in the absence of changes to DNA sequence) silencing of genes is most commonly associated with methylation of cytosine at CpG dinucleotides in gene promoter regions (DNA methylation). However, heritable repression mechanisms involving covalent modifications of histones can precede DNA methylation at gene promoters. The best characterized of these modifications involves the polycomb system and repression of transcription by the polycomb repressive complexes 1 and 2 (PRC1 & 2). PRC2 mediates repression through the histone methyltransferase activity of the component EZH2 that catalyses the trimethylation of histone H3 lysine 27 (H3K27me3). The non-catalytic core subunits of PRC2 are SUZ12, EED and RbAp46/48, but PRC2 has no obvious DNA sequence-binding capacity so in most cases it is unclear how the complex is targeted to specific promoters in mammalian cells. H3K27me3 can result in the binding of a second complex, PRC1, which – together with PRC2 – greatly increases the chances of the more stable DNA methylation mark being deposited in the development of cancer (reviewed in [7], [8]).

Epstein-Barr virus (EBV) is a gamma-herpes virus that latently infects B cells, persistently infects >90% of adult humans, causes infectious mononucleosis in some adolescents and is etiologically linked to B cell malignancies such as Burkitt lymphoma (BL), Hodgkin lymphoma (HL) and diffuse large B cell lymphoma (DLBCL). Infection with EBV can also induce the continuous proliferation (known as ‘transformation’ or ‘immortalization’) of primary human B cells *in vitro*. The lymphoblastoid cell lines (LCLs) produced in this way carry the viral genome as extra-chromosomal episomes and express only nine latency-associated proteins [six nuclear (EBNAs 1, 2, 3A, 3B, 3C & LP) and three membrane-associated (LMP1, LMP2A & 2B)] along with several RNA species (reviewed in [9], [10]). The cells are described as being latently infected because the replication of the virus and production of new virions are restricted. The latency-associated gene products are responsible for activating quiescent B cells into the cell cycle, inducing and sustaining their proliferation and maintaining the extrachromosomal episome in these latently infected cells.

*EBNA3A*, *EBNA3B and EBNA3C* are considered to comprise a family of non-redundant EBV genes which probably arose during primate gamma-herpesvirus evolution by a series of gene duplication events since they have the same gene structure (ie a short 5′ coding exon and a long 3′ coding exon), are arranged in tandem in the EBV genome and share very limited amino acid sequence homology. The EBNA3 proteins (3A, 3B and 3C) show no significant similarities to known cell or viral factors, and although none appears to bind DNA directly, they all bind the cellular DNA-binding factor CBF-1 (aka RBP-JK). All three EBNA3s can also interact with cellular factors associated with the covalent modification of histones, the repression of transcription and gene silencing. For example, EBNA3A and EBNA3C associate with histone deacetylases (HDACs), BMI1, RING1 and CtBP, ([9]–[12] and our unpublished data). EBNA3A, EBNA3B and EBNA3C are all robust repressors of transcription when targeted directly to DNA in transient assays, and EBNA3A and EBNA3C – but not EBNA3B – are necessary to establish LCLs efficiently (reviewed in [10]). EBNA3A and EBNA3C also cooperate with oncogenic Ha-Ras in the transformation/immortalization of primary rodent cells. Since this assay demonstrates a capacity to override oncogene-induced premature senescence, this indicates that EBNAs 3A and 3C may play a similar role in B cells [11]–[13].

Several reports suggest that EBNA3C can directly associate with multiple factors involved in the regulation of cell cycle progression, particularly through the G1-S transition. These proteins include the tumour suppressor pRb, the ubiquitin ligase SCF^SKP2^, cyclin D1, cyclin A, c-MYC, MDM2, p53, CHK2 and E2F1 [14]–[22]. It remains to be determined which of these interactions occurs in latently infected B cells and whether they are functionally significant.

Recent analysis suggests that together the EBNA3s can regulate >1000 host genes in B cells, and that EBNA3A and EBNA3C act as oncoproteins, whereas EBNA3B behaves as a tumour suppressor. The regulation of many of these genes appears to utilize the host polycomb system of epigenetic repression and this probably contributes to B lymphomagenesis induced by EBV [23]–[31].

Genes repressed by the combined action of EBNA3A and EBNA3C include the pro-apoptotic BH3-only protein BIM and the cyclin-dependent kinase inhibitor p16^INK4a^. EBNAs 3A and 3C are together necessary to trigger the recruitment of PRC2 core subunits and the deposition of the H3K27me3 epigenetic mark on the promoters of both of these tumour suppressor genes [23]–[25], [28], [32]. The activation mark H3K4me3 is largely unaltered at these loci irrespective of H3K27me3 status, suggesting the establishment of so-called ‘bivalent’ chromatin domains [7], [8], [33]. B cells carrying EBV encoding a conditional EBNA3C-estrogen receptor (ER)-fusion protein (EBNA3C-ER) revealed that this polycomb-mediated epigenetic repression of BIM and p16^INK4*a*^ expression is reversible, but the precise kinetics and molecular details of this have yet to be investigated [25], [28]

In order to determine the relative importance of the epigenetic repression of *INK4a* in B cell transformation and LCL outgrowth, we made use of primary B cells from an individual with a genetic lesion that prevents expression of functional p16^INK4a^, together with the conditional EBNA3C-expressing virus. We have also explored the early events following infection and outgrowth of LCLs from normal p16^INK4a^-expressing B cells.

## Results

### Production and validation of LCLs that are functionally null for p16^INK4a^

**Figure 1:**
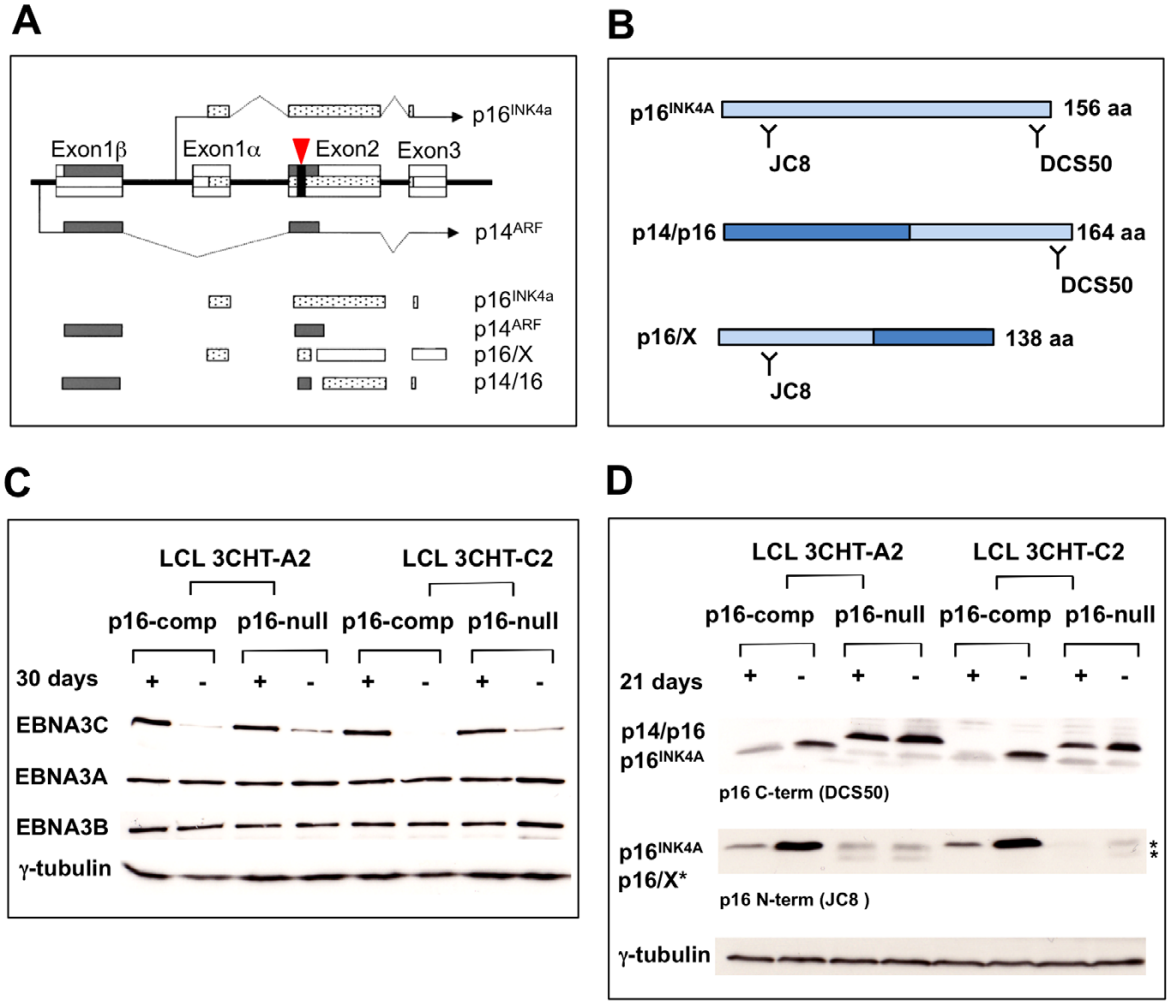
Characterization of Leiden LCLs with conditional EBNA3C. (**A**) *CDNK2A* transcripts in normal cells and in individual with a 19bp deletion in the gene (reproduced from [36]). The coding sequence of p16^INK4a^ is shown in stippled boxes, p14^ARF^-encoding sequence in grey boxes, the deletion is shown as a black box highlighted by a vertical arrow. Normal p16^INK4a^ and p14^ARF^ transcripts are depicted above the two chimerical transcripts p16/X and p14/p16. (**B**) Schematic representation of the WT p16^INK4a^ protein produced in p16-competent LCLs and the two chimeric proteins produced in p16-null LCL 3CHT lines. The epitopes detected by both JC8 and DCS50 anti-p16^INK4a^ antibodies are depicted. (**C**) Expression of EBV latent proteins in p16-null and p16-competent LCL 3CHT with or without HT. p16-competent lines LCL 3CHT-A2 and LCL 3CHT-C2 and p16-null LCL 3CHT-A2 LCL 3CHT-C2 were LCL cultured for 30 days without 4HT, proteins extracted, subjected to SDS-PAGE and western blotted with the antibodies indicated. Only EBNA3C is degraded. Gamma-tubulin was used as a loading control. (**D**) Western blot comparing expression of the proteins arising from *CDKN2A* locus in two p16-null LCL 3CHT lines and two p16-competent LCL 3CHT lines, as used in (C), cultured 21 days with (+) or without (−) 4HT. The p16/X protein doublet is indicated by asterisks. Data are representative of two independent experiments including four p16-null and two p16-competent LCL 3CHT lines.

To establish LCLs from primary B cells that are incapable of expressing functional p16^INK4a^, we made use of peripheral blood leukocytes (PBL) from an individual who is homozygous for a 19-base pair germ-line deletion in the second exon of the *CDKN2A* gene ([34]–[36]; Figure 1A). Since exon 2 is shared by both p16^INK4a^ and p14^ARF^, neither of the wild type proteins is expressed. However two chimeric polypeptides originate from the disrupted locus: chimera p16/X consists of a fusion of the first 74 residues of p16^INK4a^ with 64 amino acids that are specified by the +1 reading frame of exon 2, whereas the p14/p16 chimera has the amino terminal 88 residues of p14^ARF^ fused in frame to the last 76 residues of p16^INK4a^. The p14/16 fusion retains all known functions of p14^ARF^, but neither protein exhibits any of the known functions of p16^INK4a^ ([35], [36]; Figure 1B).

PBL carrying this deletion (sometimes called the Leiden deletion) were infected with recombinant BAC-based B95.8 EBV expressing the EBNA3C-modified estrogen receptor fusion (3CHT) described previously [25]. After infection, cells were cultured in the presence of the activating ligand 4-hydroxytamoxifen (4HT) to ensure the outgrowth of infected B cells expressing the active EBNA3C fusion. Four LCLs were established using each of two independently derived 3CHT viruses (LCL 3CHT-A1, -A2, -C1, -C2). LCLs produced with Leiden B cells and carrying a conditional EBNA3C were called LCL 3CHT p16-null. LCLs produced using the same viruses and normal B cells from a healthy blood donor were called LCL 3CHT p16-competent. Once LCLs were established (generally 2–3 months post-infection), aliquots of cells were taken and diluted in fresh medium; one was then continuously grown with 4HT (+4HT) and the other grown without (−4HT). After at least 3 weeks, proteins extracted from the LCLs were separated by SDS-PAGE and analyzed by western blotting to show EBV proteins EBNA3A, EBNA3B and EBNA3C or p16^INK4a^-related polypeptides (see for example Figure 1C and D). Irrespective of whether the cells were p16-competent or p16-null, in the absence of 4HT the EBNA3C protein is inactivated by sequestration from the nucleus and degradation, hence less protein was detected (Figure 1C). As has been described previously, no consistent change in the expression of other EBV latency-associated proteins was detected ([25], [37]; data not shown and see below). The pattern of p16^INK4a^ related fusion proteins expressed was similar to that described previously in Leiden fibroblasts and LCLs established by infecting Leiden B cells with the B95.8 strain of EBV [35], [36]. In the LCL 3CHT expression of both p16^INK4a^ and p14/p16 was induced by removal of 4HT and the non-functional p16/X fusion, although difficult to detect, appeared as a polypeptide doublet (Figure 1D and also [35]).

### The *CDKN2A* locus is still epigenetically regulated by EBNA3C in the absence of functional p16^INK4a^

As outlined in the Introduction, EBNA3C – by cooperating with EBNA3A – functions to repress transcription of p16^INK4a^ in infected B cells, by facilitating the recruitment of polycomb complexes to the *CDKN2A* locus and the deposition of the epigenetic repressive mark H3K27me3 upstream of the *INK4a* transcription start site [25]. Moreover, EBV does not appear to significantly alter the amount of H3K4me3 at the locus, suggesting the promoter is bivalent in B cells as has been described in various other types of cell including fibroblasts and embryonic stem cells [33].

**Figure 2:**
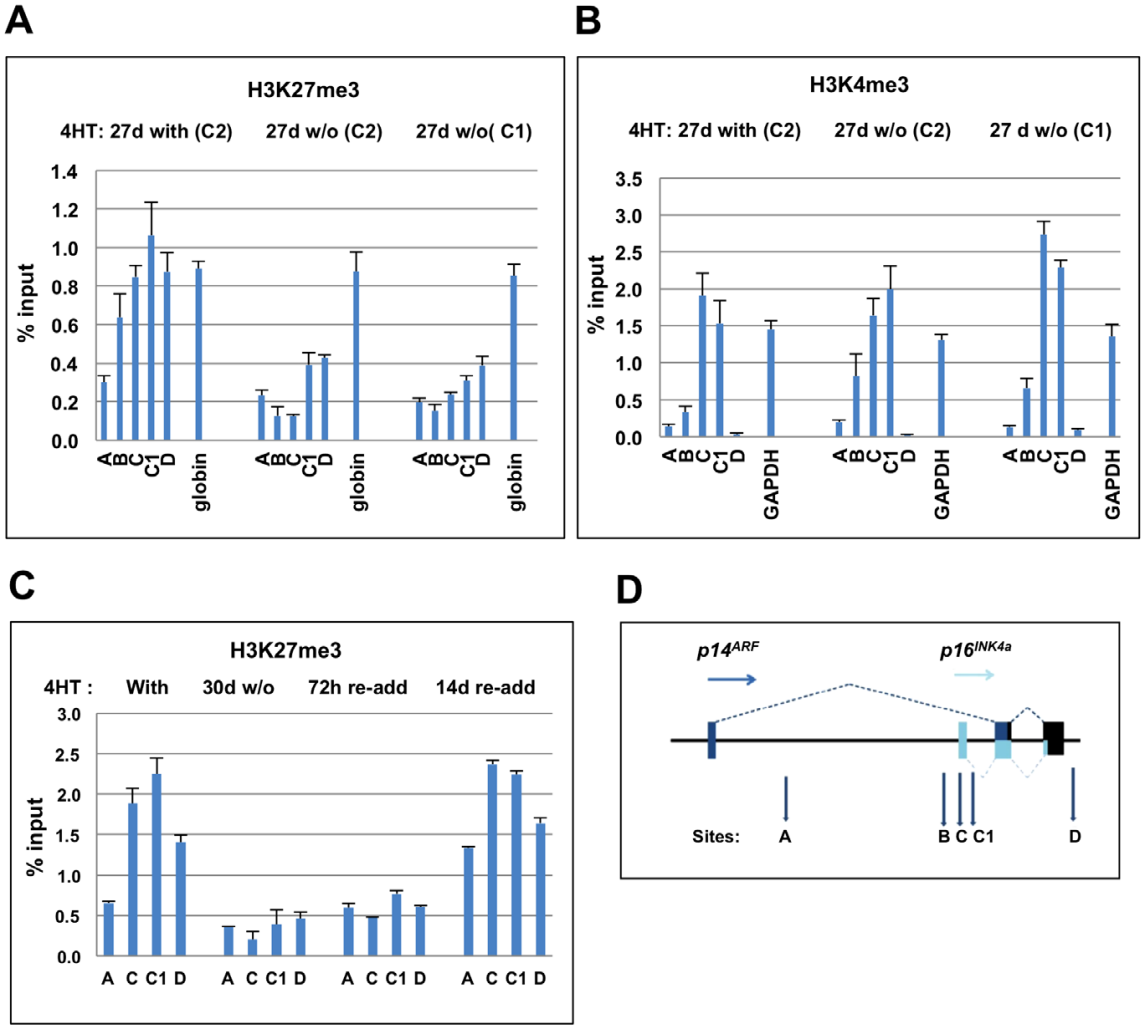
Epigenetic regulation of the *INK4A* locus by EBNA3C in p16-null LCL 3CHT cells. (**A**) ChIP-qPCR analysis quantifying the H3K27me3 across *CDKN2A* locus in two p16-null LCL 3CHT lines (corresponding to C1 and C2 in Figure 1) cultured 27 days with or without 4HT. The peak of H3K27me3, centered at *INK4a* exon 1, is detected in the p16-null LCL 3CHT cells cultured with 4HT and is substantially diminished in the lines cultured without 4HT. H3K27me3 mark at the silenced gamma-globulin promoter remains stable regardless of EBNA3C activity. (**B**) Similar ChIP-qPCR analysis quantifying the H3K4me3 across *CDKN2A* locus in p16-null LCL 3CHT cells. The peaks of H3K4me3 centered at *INK4a* exon 1 that were detected in two p16-null LCL 3CHT lines (C1 and C2) cultured for 27 days with or without 4HT do not significantly differ, irrespective of whether 4HT is present and EBNA3C is active. H3K4me3 at the active *GAPDH* promoter serves as a positive control. (**C**) The kinetics of epigenetic modulation of *INK4a* locus by EBNA3C: ChIP-qPCR analysis quantifying the H3K27me3 mark across the *CDKN2A* locus in p16-null LCL 3CHT cells (A2) cultured with 4HT, 30 days without 4HT or 72 h and 14 days after 4HT was re-added. The peak of H3K27me3 centred at *INK4a* exon 1 that was detected in p16-null LCL 3CHT cultured with HT is diminished in lines cultured without HT and is gradually restored following the re-addition of 4HT. (**D**) A schematic of the *CDKN2A* locus depicting the location of the assays used in ChIP-qPCR. The details of the primer position and sequence are described in [25].

Chromatin immunoprecipitations, coupled with quantitative PCR (qPCR) assays directed across the *CDKN2A* locus, were performed on the LCL 3CHT p16-null cells cultured plus or minus 4HT in order to establish whether EBNA3C was functional and regulated by 4HT. Although the p16^INK4a^ fusion proteins retain no p16^INK4a^ function, EBNA3C in the p16-null LCLs promotes similar chromatin modifications to the *CDKN2A* locus as previously shown for p16-competent cells, that is the deposition of H3K27me3 mark across the p16^INK4a^ promoter region while it remains marked with H3K4me3 (compare Figure 2 A and B with [25]). This confirms that the chromatin modifications induced at this locus are not dependent on p16^INK4a^ functioning as an inhibitor of cyclin-dependent kinases and that the transcriptional reprogramming activity of EBNA3C can be switched on and off in these cells (Figure 2C).

The fact that EBNA3C still regulates expression of the *INK4a* locus, when proliferative advantage of the EBNA3C-expressing cells is unlikely to be a contributory factor, provides further evidence that EBNA3C (and EBNA3A) might directly target the *CDKN2A* locus rather than the epigenetic changes being a secondary consequence of selection. In order to test this, we made use of recombinant EBV-BAC expressing epitope-tagged EBNA3C [28]. LCLs were established with this tagged virus and with un-tagged wild-type EBV-BAC using cells from the same donor. Anti-FLAG antibodies were used to perform ChIP assays from these LCLs and qPCR primer sets corresponding to sites across the *INK4b/ARF/INK4a* locus were used to analyse the precipitated DNA (Figure S1). The histograms show that EBNA3C is targeted to regions proximal to not only the *ARF* and *INK4a* transcription start sites (TSS), but also the *INK4b* TSS. These data are consistent with EBNA3C (alongside EBNA3A) associating with chromatin at several sites and regulating the polycomb-mediated repression of the whole *INK4b/ARF/INK4a* locus. Similar coordinated polycomb-mediated repression of this locus has been reported in other systems (reviewed in [1], [5]). The basis of EBNA3C targeting and the nature of the coordination are presently unknown, but these ChIP results add considerable weight to the hypothesis that EBNA3C (with EBNA3A) acts directly on chromatin at 9p21, rather than indirectly through a signaling pathway.

### Inactivation of EBNA3C alters neither proliferation nor Rb-E2F function in p16-null LCLs

**Figure 3:**
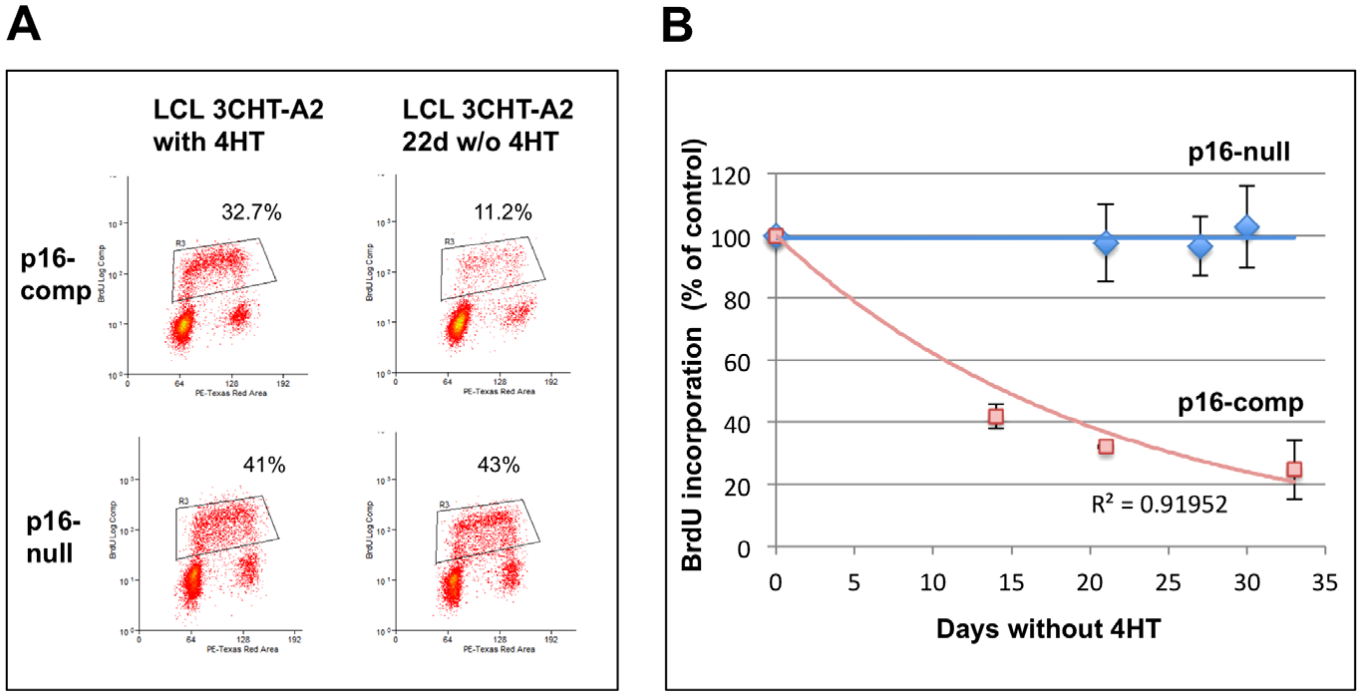
Proliferation rate of p16-null and -competent LCL 3CHT lines following EBNA3C inactivation. (**A**) Representative FACS analysis of cells cultured for 22 days with or without 4HT, then incubated for 1 hour with BrdU and stained with anti-BrdU-FITC/PI. The gated population comprises the cells that have undergone DNA replication while incubated with BrdU (cells that have DNA content between 2N and 4N and highly incorporate BrdU). (**B**) BrdU incorporation in every cell line cultured without 4HT was normalized at each time-point to values measured in the respective cell line cultured with 4HT. Comparison of the proliferation of four p16-null LCL 3CHT lines (C1, C2, A1, A2; shown as diamonds) and two p16-competent LCL 3CHT lines [-C and -A, (line A in duplicate experiments); shown as squares], quantified by BrdU incorporation, in the time-courses of up to 30 days following removal of 4HT. The time interval between sample harvesting sometime differed between lines. The proliferation rate of the p16-competent LCL 3CHT lines cultured without 4HT progressively declines with time, while the proliferation of the p16-null LCL 3CHT lines without 4HT remains unaltered. Error bars indicate the standard deviations between experiments.

Having established that in the presence of the activating ligand 4HT the conditional EBNA3C remains capable of acting as an epigenetic modifier, we explored its role in sustaining LCL proliferation. Initially p16-competent and p16-null LCL 3CHT lines that had been established with 4HT were split, half were cultured without 4HT and half with 4HT for the next 22 days. Aliquots were taken and cultured with the thymidine analogue BrdU for 1 hour, harvested, stained with anti-BrdU-FITC and propidium iodide and analyzed by flow cytometry to reveal the percentage of cells undergoing DNA synthesis – and therefore proliferating – during the 1 hour BrdU-pulse. A representative experiment is shown in Figure 3A. It is clear that when EBNA3C is inactive, the LCL with wild type p16^INK4a^ incorporates substantially less BrdU, consistent with the cell cycle arrest described in previous reports [25], [37]. In contrast the p16-null cells continue to proliferate at a similar rate in the absence of 4HT as when 4HT is retained in the medium and EBNA3C is functional. A comparison of all four p16-null lines with two p16-competent lines cultured for up to 30 days without 4HT and analyzed by the same BrdU-pulse strategy confirmed and extended these results (Figure 3B).

**Figure 4:**
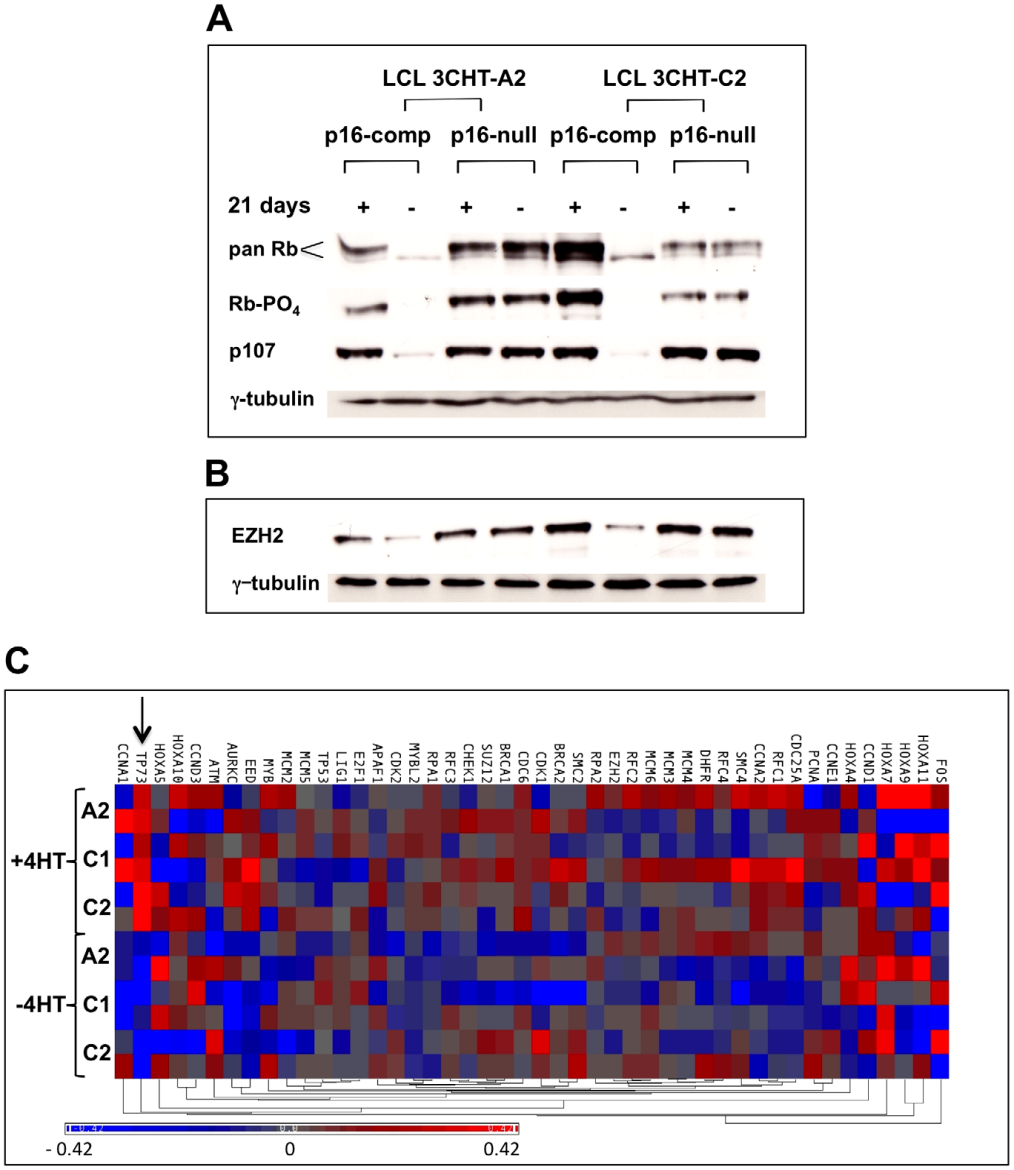
Quantity and phosphorylation state of the ‘pocket’ proteins and expression of E2F1-target genes in p16-null and -competent LCL 3CHT lines with or without 4HT. (**A**) Similar cell extracts to those used in Figure 1D were separated by SDS-PAGE and western blotted using a pan-specific anti-Rb antibody (pan-Rb) to detect all forms of Rb from hypophosphorylated (lower bands) to hyperphosphorylated (upper bands). Anti-phospho-Rb antibody (Rb-PO_4_) detects only the hyperphosphorylated forms of Rb and anti-p107 indicates the pocket protein p107. Gamma-tubulin was used as a loading control. In p16-competent LCL 3CHT lines cultured for 21 days without 4HT, Rb is down-regulated and hypophosphorylated and p107 is also down-regulated. In p16-null LCL 3CHT lines (A2 and C2 in Figure 1), no difference in the quantity of Rb or p107 nor Rb phosphorylation is detectable regardless of EBNA3C activity. (**B**) Regulation of the E2F1-target EZH2. Samples of protein extracts used in (A) were separated by SDS-PAGE and western blotted with an antibody specific for the polycomb complex subunit EZH2 – a protein induced by E2F1. Again gamma-tubulin was used as a loading control. All these data are representative of two independent experiments each including at least two p16-null LCL 3CHT lines. (**C**) Heat-map visualization of microarray data from p16^INK4a^-null 3CHT LCLs grown in the presence or absence of 4HT for more than 30 days. This displays relative transcript levels (i.e. raw transcript levels corrected for variation between the three cell clones and then mean-normalized for each gene) for E2F1 and 45 of its transcriptional targets. The scale indicates high (red) to low (blue) changes in transcript levels, with the extremes of the scale indicating a 0.42-fold change in expression (ie a sample on the extreme red end of the scale would show a 1.42-fold expression increase over one at the center - grey). The only gene significantly and consistently altered in its expression by the inactivation of EBNA3C is TP73 (indicated by arrow), which falls upon EBNA3C inactivation. The dendrogram (bottom) shows the ordering of the genes by their clustering into co-regulated groups. Cell line clone IDs and treatment (±4HT) are indicated to the left.

Consistent with the DNA synthesis data, in the p16-competent LCLs inactivation of EBNA3C resulted in a predictable dephosphorylation of Rb and down-regulation of p107 (Figure 4A and [25]). In contrast no changes in the phosphorylation status or quantity of these ‘pocket’ proteins were detected in the p16-null LCLs when 4HT was removed and EBNA3C inactivated (Figure 4A). Moreover, in the p16-competent LCLs the lack of functional EBNA3C caused a reduction in the expression of an E2F1-target gene EZH2 [38], but in the p16-null cells EBNA3C had no effect on EZH2 expression (Figure 4B). Although this result is consistent with the status of Rb in these cells (ie whether or not it is activated by dephosphorylation) it was perhaps surprising, since EBNA3C has recently been reported to directly alter E2F1 activity by suppressing its expression and inhibiting its capacity to transactivate target genes [22].

### Exon microarray analysis reveals EBNA3C-regulated genes in continuously proliferating p16-null LCLs

In order to determine whether EBNA3C alters the expression of other known E2F1-regulated genes in p16-null cells, a genome-wide microarray approach was adopted. This had the added value of identifying genes regulated by EBNA3C in non-tumour B cells – independently of p16^INK4a^, the status of the pRb-E2F1 axis and cell proliferation.

To investigate the effect of EBNA3C on the transcriptome of EBV-infected p16-null cells, 4HT was withdrawn from and re-added to three 3CHT cell lines established in the background of the Leiden p16^INK4a^ mutation (as described in Materials and Methods and Figure S2). Gene level analysis identified 429 differentially regulated genes (p value<0.001) of which 252 have a fold change >1.4, and 82 have changed more than two-fold (Table S1). This analysis identifies some previously reported [29] EBNA3C-repressed genes (for example *ADAMDEC1* and *ADAM28*) among those exhibiting the greatest degree of de-repression upon inactivation of EBNA3C, while another – encoding the tumour suppressor BIM (*BCL2L11*) – is altered in expression (p = 0.0005) but only to a modest degree (1.58-fold). *CDKN2A*, despite the observations made in this paper, is not on this list (p = 0.0012; fold change = 1.40 just fails the cut-off used). The raw data indicate that this low statistical confidence is because one of the three cell lines appears to have lost the ability to repress the mutated *CDKN2A* locus (Figure S3). EBNA3C up-regulates 39 genes at least two-fold, including *AICDA* – the gene encoding the cytidine deaminase that is responsible for mutation of immunoglobulin variable regions in germinal center B cells [39].

Since EBNA3C has been reported to target and repress E2F1 transcriptional activity [22], we specifically reviewed E2F1 and E2F-target genes. In keeping with our observations that the dependence of cell proliferation on EBNA3C requires the action of p16^INK4a^, analysis of E2F1 and a set of forty-five E2F1-target genes (based on those described in [40]) failed to show any differential expression attributable to inhibition of E2F1, between cells with active or inactive EBNA3C (Figure 4C). The only differentially regulated gene (p<0.001; fold change >1.4) from this list was *TP73* (indicated in Figure 4C), which was modestly (1.7-fold) but consistently down-regulated upon EBNA3C inactivation (p = 7×10^−5^). This is the opposite direction to that expected from a mechanism involving the inhibition of E2F1 by EBNA3C [22]. Taken together, these data suggest that in the absence of p16^INK4a^, EBNA3C has no effect on the regulation of E2F1 or E2F1-target genes.

### The p53-p21^WAF1^ pathway is not affected by loss of EBNA3C function in p16^INK4a^-null LCLs

**Figure 5:**
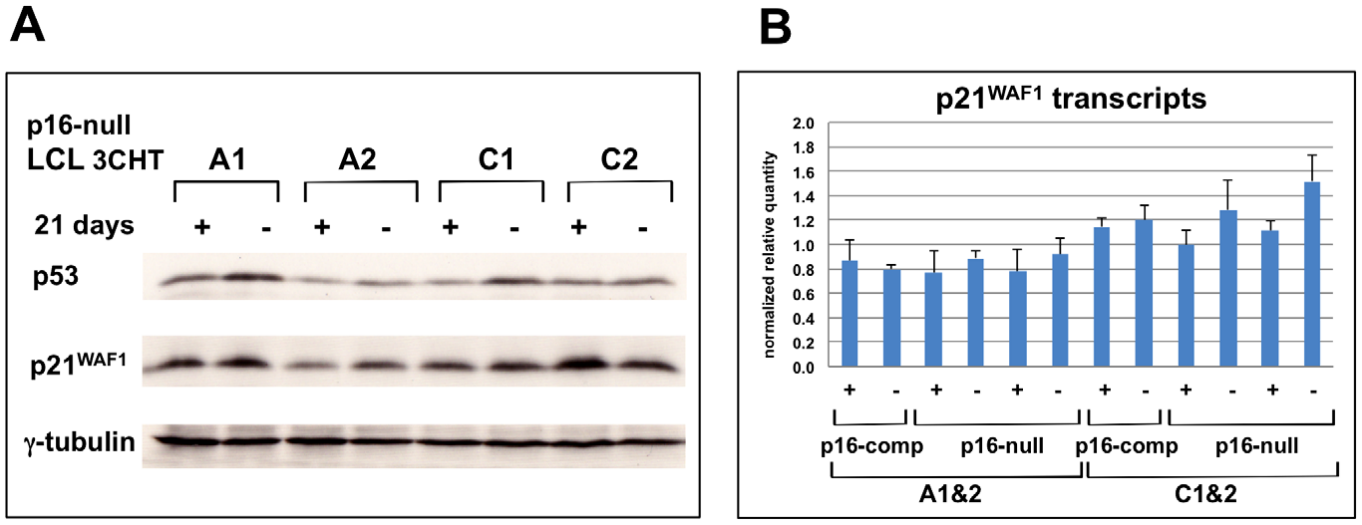
p53-p21^WAF1^ pathway is not activated in p16-null LCL 3CHT cells following removal of 4HT and inactivation of EBNA3C. (**A**) Four p16-null LCL 3CHT lines were cultured for 21 days with (+) or without (−) 4HT. Protein extracts were separated by SDS-PAGE and western blotted for p53 or p21^WAF1^. No consistent changes in expression of p53 or p21^WAF1^ were observed. (**B**) qPCR of cDNAs corresponding to p21^WAF1^ transcripts in the same four p16-null LCL 3CHT lines (A1, A2, C1, C2) and two p16-competent LCL 3CHT lines (A and C) cultured for 21 days with or without 4HT. The data are normalized to the expression of control RNA.

The p14/p16 chimeric protein expressed in p16-null Leiden fibroblasts has been extensively characterized and shown to retain all known functions of p14^ARF^ [35], [36]. This chimera was modestly, but consistently up-regulated in p16-null LCL 3CHT cells upon inactivation of EBNA3C by removal of 4HT (Figure 1D). In order to determine whether there were any downstream consequences of this increase, cell extracts were analyzed by western blotting for p53 – the main functional target of p14^ARF^, and for p21^WAF1^ – a transcriptional target of p53 [1], [3]. Although the levels of p53 and p21^WAF1^ protein varied slightly between cell lines and experiments, there was no consistent accumulation of either in response to EBNA3C inactivation (see for example Figure 5A). Moreover there was no significant increase in p21^WAF1^ RNA detected by quantitative RT-PCR (Figure 5B). These data are consistent with the cells still proliferating in the absence of 4HT and suggest that p14/16 has no significant anti-proliferative activity in this context. Similar results were obtained when the p14^ARF^-p53-p21^WAF1^ pathway was analysed in p16^INK4a^-competent LCL 3CHT, ie there was evidence of slight de-repression of the *ARF* locus, and a marginal increase in p14^ARF^ was apparent when EBNA3C was inactivated (Lenka Skalska PhD thesis, Imperial College London).

### EBNA3C has no impact on p53 or p21^WAF1^ function after DNA damage in LCLs, irrespective of their p16^INK4a^ status

**Figure 6:**
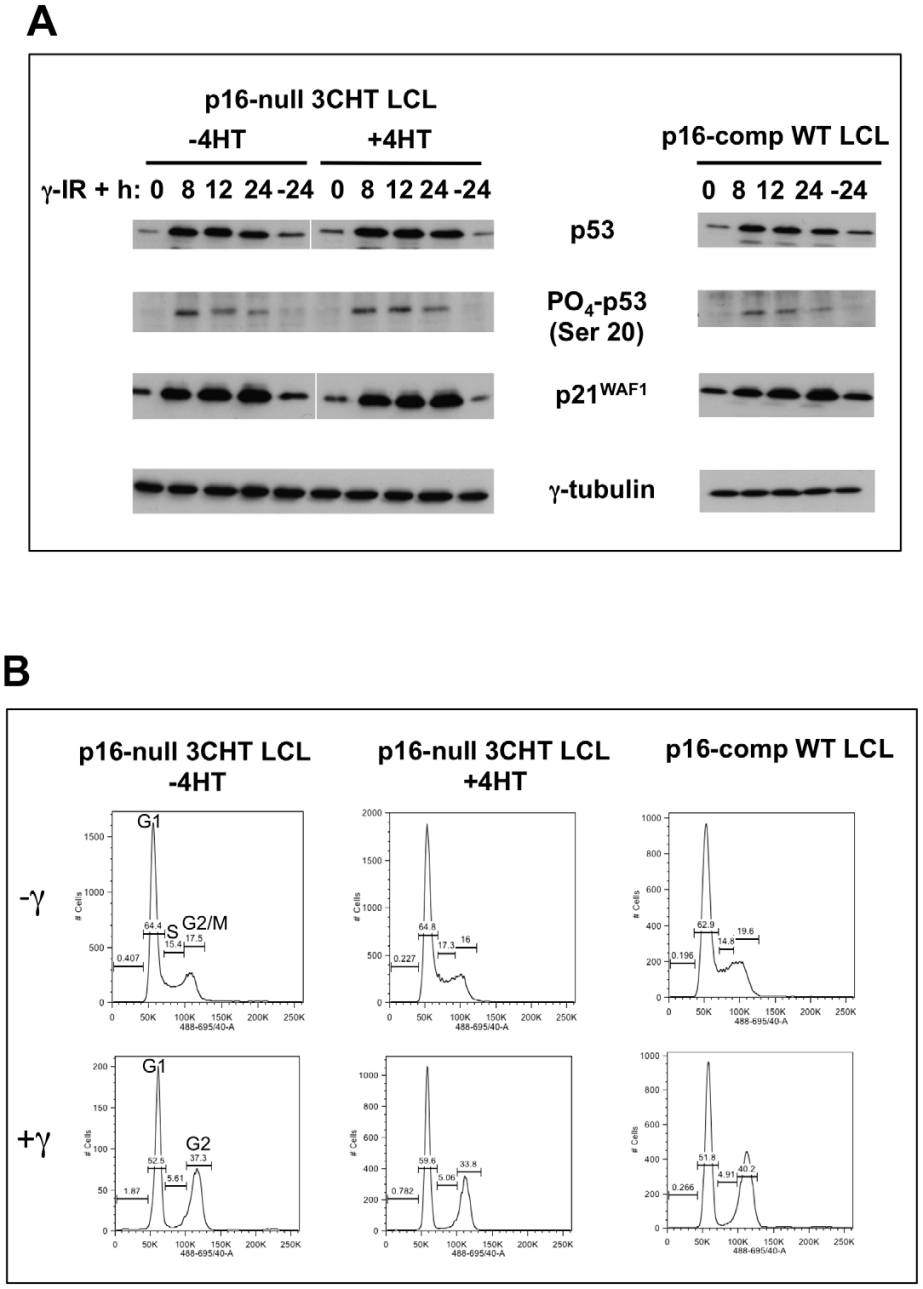
EBNA3C has no impact on the response of LCLs to γ-IR, irrespective of their p16^INK4a^ status. (**A**) Western blots showing the levels of p53, phospho-p53 (Ser 20), and p21^WAF1^ from LCLs (3CHT LCL-C2 and a wild-type EBV-BAC LCL) exposed to 8.5 Gy (800 rad) γ-IR and left for the times indicated. Gamma-tubulin is shown as a loading control. (**B**) Samples of the cultures from which proteins were western blotted in A, were fixed, stained with PI and analysed by flow cytometry.

It has been suggested that EBNA3C might target p53 directly and indirectly – via MDM2, Gemin3 and ING4/5 – to impair its function [18], [19], [21], [41]. Since in p16-null LCLs there was an increase in the p14/p16 fusion protein after inactivation of EBNA3C, but no consistent activation of the p53-p21^WAF1^ pathway or reduction in proliferation, we asked whether the pathway remained intact in these cells. This was achieved by determining whether EBNA3C had any effect on the response of these LCLs to DNA damage induced by gamma-irradiation (γ-IR), since this is a well established activator of the pathway ([42]; reviewed in [43]). The results show clearly that both p16-competent and p16-null LCLs exposed to γ-IR respond with the phosphorylation of the CHK1/2 target on p53 (serine 20, [44]), p53 stabilization and activation, the accumulation of p21^WAF1^ and concomitant cell cycle arrest (Figure 6A and B). This was irrespective of whether EBNA3C is functional. Similar results were obtained with a second independent pair of 3CHT LCLs (data not shown). These data suggest that in the context of latent infection of B cells, EBNA3C has no detectable effect on p53 activation, its ability to transactivate *p21^WAF1^* and induce cell cycle arrest. The data presented here are consistent with the behavior of ‘normal’ LCLs established with the B95.8 strain of EBV and also mitogen activated primary B cells [42].

Since the p53-p21^WAF1^ pathway appeared to be intact, and the p16^INK4a^-null cells were capable of G1 and G2 cell cycle arrest following DNA damage, we wanted to show in an independent assay that Rb could be dephosphorylated and the cells could arrest in G1. When ‘normal’ p16-competent LCLs are grown to high density, the majority of the cells arrest in G1 and Rb is present only in its hypophosphorylated, activated form [45]. The two p16-null 3CHT LCLs analysed for p53/p21 function were therefore grown to saturation density (5 days in culture without a change of medium) to establish whether Rb could be activated by dephosphorylation in these cells. The results (see for example Figure S4) showed that at high density Rb is dephosphorylated and cells accumulate in G1, irrespective of either the p16^INK4a^ or EBNA3C status of the LCLs. These data demonstrate unequivocally that Rb – the operational target of p16^INK4a^ – can be activated in the Leiden LCLs and that EBNA3C does not override a p16^INK4a^-independent G1 arrest.

### Expression of *CDKN2A* transcripts during the transformation of normal primary B cells

All the data described above are consistent with p16^INK4a^ being the major barrier to proliferation of established LCLs; EBNA3C (cooperating with EBNA3A) epigenetically represses transcription from *CDKN2A* in order to prevent the cell cycle arrest induced by p16^INK4a^. However, soon after the infection of resting B cells, when they are activated to become B-blast-like and driven into the cell cycle the nuclear environment is very different to that in the transformed, continuously proliferating LCL cells. Furthermore these LCL populations may have already become oligoclonal or even a single clone that has been selected by the *in vitro* culture conditions [46]. It was therefore important to establish how p16^INK4a^ was regulated in newly infected primary B cells.

**Figure 7:**
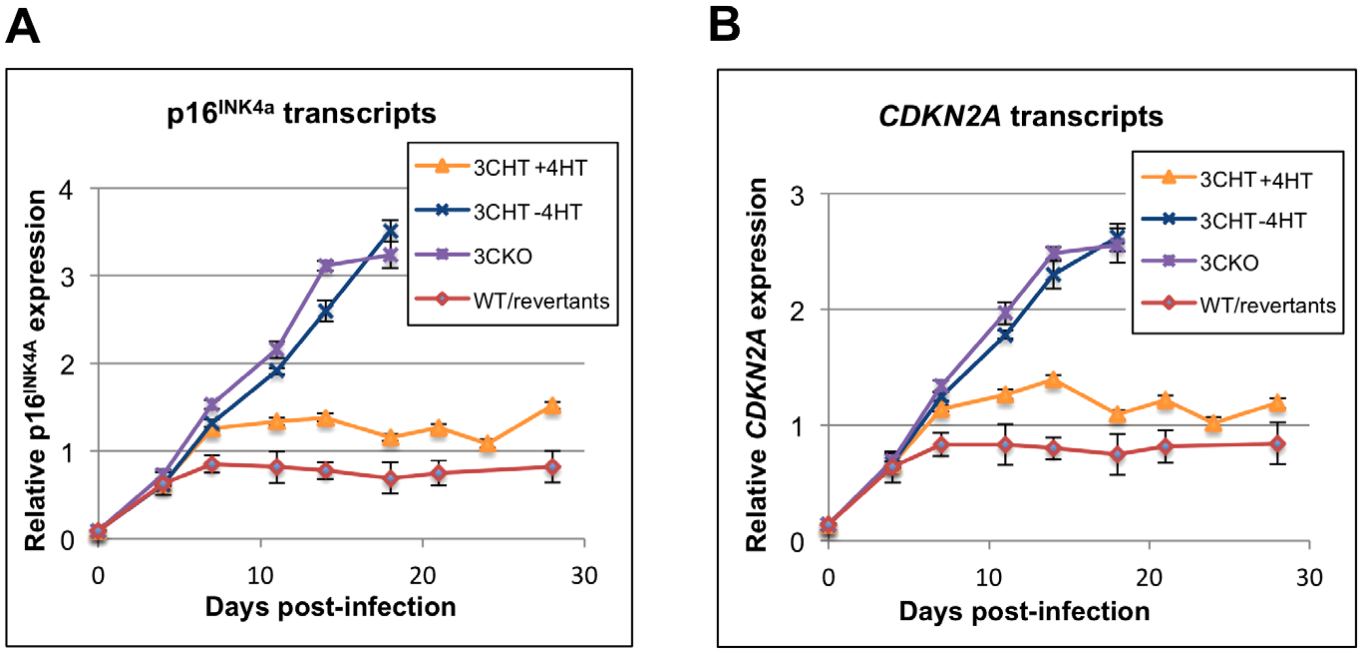
Quantitative PCR analysis of *CDKN2A* transcript levels after primary infection of B cells. B cells combined from four blood donors were isolated using anti-CD19 magnetic beads and infected by the viruses indicated at day 0. Zero time point is RNA from the cells prior to infection. WT/revertant data shows the mean of infection by four viruses (WT, 3C-revertant, 3A-revertant and E3-revertant viruses). Graphs show the same cDNA analyzed with an assay within *INK4a* exon 1 (**A**) and one between the common exons 2 and 3 (*CDKN2A*) shared by p16^INK4a^ and p14^ARF^ transcripts (**B**). Expression data were normalized to expression of *ALAS1*, *GNB2L1* and *RPLP0* and expression values are expressed relative to the average of all data points.

In order to investigate the quantity of p16^INK4a^-encoding transcripts expressed in normal primary B cells during the first weeks after infection with EBV, two RT-PCR assays were utilized – one based on a qPCR detecting an amplicon in *INK4a* exon 1 (Figure 7A) and a second that targets the exon 2/3 boundary shared by p16^INK4a^ and p14^ARF^ (Figure 7B). Both assays showed that *INK4a/CDKN2A* is silent or has very low activity in purified CD19-positive, resting B cells at the time they are infected. Nevertheless, irrespective of whether or not the infecting virus expresses functional EBNA3C (WT-BAC, EBNA3C-revertant or 3CHT plus 4HT) or is EBNA3C-deficient (EBNA3CKO or 3CHT minus 4HT), during the first few days post-infection there is a substantial increase in transcripts corresponding to *INK4a/CDKN2A*. This is the period that corresponds to EBNA2 and EBNA-LP expression, blast activation, synthesis of cyclin D2, synthesis of c-MYC and rapid cell proliferation [47]–[50]. However, 4–7 days post-infection the effect of EBNA3C was seen. In cells infected with wild type (WT-BAC) or EBNA3C-revertant virus, expression of *INK4a/CDKN2A* no longer increases and remains relatively constant – presumably below a critical threshold – up to the end of the experiment at 28 days. In contrast, in cells infected with EBNA3CKO or 3CHT without 4HT, *INK4a/CDKN2A* transcript levels rise progressively until at least 18 days. At, and beyond this time, there was substantial cell death in the EBNA3C-deficient cell populations (see below) such that beyond 18 days, it was not possible to isolate sufficient RNA for analysis. Consequently no samples from EBNA3C-deficient cell lines were assayed for *INK4a/CDKN2A* transcription after day 18.

When B cells were infected with the 3CHT virus and then cultured in 4HT, the amount of *p16^INK4a^/CDKN2A* RNA reached a steady state, with similar kinetics, but at a slightly higher level than in wild type and revertant-infected cells (Figure 7A and B). These data are consistent with our original observations that the EBNA3C-estrogen receptor fusion protein is a little less efficient than unmodified EBNA3C at regulating the level of p16^INK4a^ protein in established LCLs, but enables proliferation [25].

Taken together, the results suggest that soon after the infection of primary B cells, EBV induces *p16^INK4a^* transcription and EBNA3C (with EBNA3A) is necessary to prevent the accumulation of p16^INK4a^ above a threshold that would block proliferation and LCL outgrowth.

### When after infection does functional EBNA3C become vital for the transformation of B cells into LCLs?

**Figure 8:**
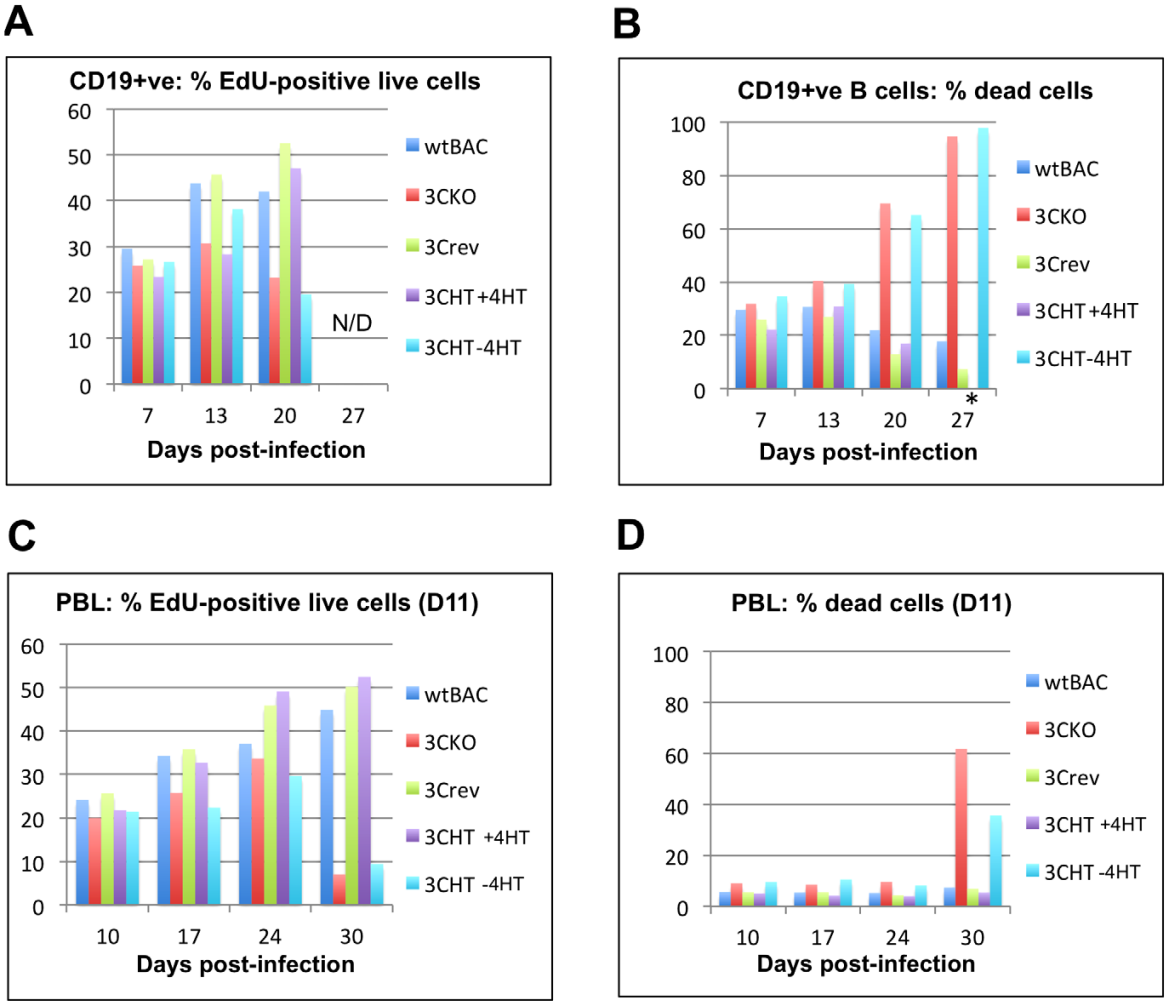
Cell proliferation and viability after primary infection of B cells. B cells isolated using anti-CD19 magnetic beads (**A and B**) or ficoll-purified bulk lymphocytes from a buffy-coat residue (from donor D11) (**C and D**) were infected by the viruses indicated, at day zero. The proportion of proliferating cells (**A and C**) was assessed by EdU incorporation over 16 hours. Cell viability was assessed by Live/Dead staining (**B and D**). The sampling time after infection, and the virus used to infect the cells are as indicated. Note that FACS statistics were not collected for 3CHT+HT at 27 days due to a technical failure during automated data acquisition (_*_). However visual inspection indicated that the majority of the cells were viable and this was consistent with the yield of RNA recovered from these cells (which was comparable to the WT and revertant infections – see figure 7).

The populations of CD19-positive B cells that were infected with EBNA3C-expressing or EBNA3C-deficient viruses and sampled for transcript analysis were also sampled at the times indicated to assess DNA synthesis (Figure 8). Samples were taken and pulsed for 16 hours with the alkyne- containing thymidine analogue EdU, labeled with AF488 dye and analysed by flow cytometry to reveal the percentage of cells incorporating EdU and therefore proliferating. A histogram (Figure 8A) and representative plots (Figure S5) show that after 7 days, 20–30% of the cells are proliferating, irrespective of whether or not EBNA3C is functional (as was shown previously [50]). By 13 days, only in the populations of cells carrying EBNA3C-expressing virus does the percentage of EdU-positive cells significantly increase and after 20 days nearly 50% of this population is EdU-positive and therefore proliferating. In contrast the proportion of EdU-positive cells in the EBNA3C-deficient populations fails to increase after 13 days and is declining by 20 days. Visual inspection of these cultures indicated substantial numbers of cells were dying. This was confirmed by flow cytometry after vital staining (Figure 8B). By 27 days more than 90% of the EBNA3C-deficient populations were dead, such that there were insufficient surviving cells to permit reliable analysis of proliferation at this time point.

In our experience purified CD19-positive B cells are less robust during outgrowth of LCLs than if one starts with PBL. This is probably because the latter contain a sub-population of non-infected viable cells such as macrophages that act as a paracrine source of B cells survival factors. The experiment described above was therefore repeated with PBL (independently with cells from two blood donors – D11 and D13) and EdU incorporation and viability were assessed at the times after infection indicated (Figure 8C and D; Figure S6). The percentages of cells proliferating followed similar trends to the CD19-positive B cells, with little difference in proliferation rates at 10 days, and the effects of EBNA3C-deficiency becoming more apparent as the infection proceeded, such that proliferation had dropped to 10% by 30 days in the EBNA3C-deficient infections (Figure 8C), with over 50% of detectable cells being dead (Figure 8D). We note that this assay does not distinguish between the remaining live EBV-infected B cells and other surviving non-B cell lineages, which might contribute to the cell culture at this time – however the proportion will be similar in each different infection. In both PBL and CD19-purified B cell experiments, no EBNA3C-deficient cell lines grew out as LCLs. These data are therefore consistent with the hypothesis that accumulating *INK4a/CDKN2A* transcripts (Figure 7) are translated into p16^INK4a^ protein that then activates pRb and induces cell cycle arrest (and/or cell death) when EBNA3C is unavailable.

### EBNA3C is dispensable for the production of p16^INK4a^-null LCLs

If p16^INK4a^ is the major target of EBNA3C and barrier to B cell transformation, a prediction is that functional EBNA3C never has to be expressed to produce p16^INK4a^-null LCLs. Leiden PBL were therefore infected with 3CHT EBV and cultured in the presence (3CHT with 4HT) and absence (3CHT never 4HT) of 4HT. Additional infections were performed with 3CKO and 3C-revertant viruses. The relatively small number of viable cells in the Leiden PBL aliquot used for this experiment prevented meaningful proliferation analysis of the cell lines during the early stages of outgrowth.

**Figure 9:**
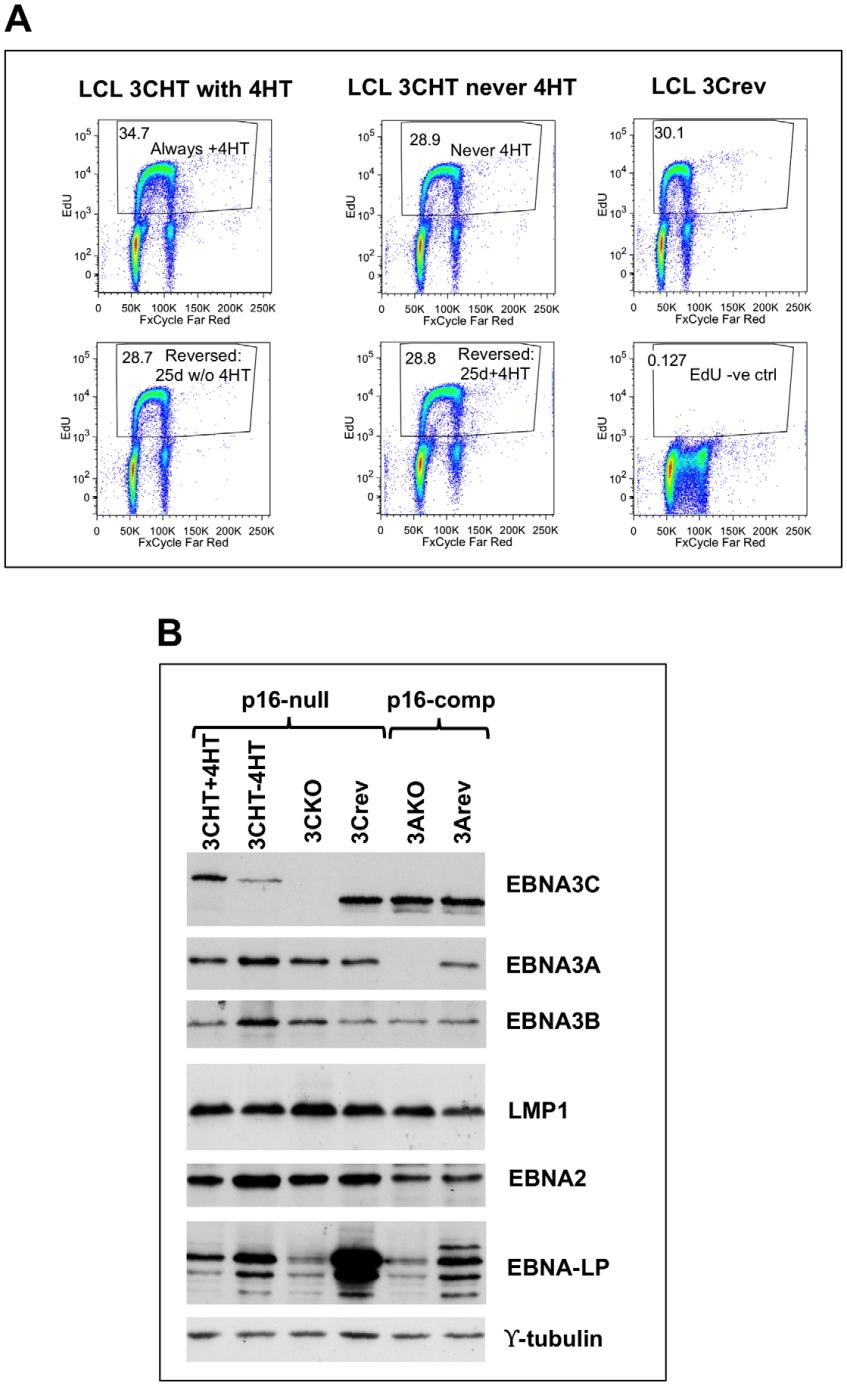
Characterization of EBNA3C-deficient LCLs established in a p16^INK4a^-null background. (**A**) Proliferation assessed by EdU incorporation (1-hour pulse) of 3CHT LCLs established in the absence (never 4HT) or presence (3CHT with 4HT) of 4HT. Cells were split and 4HT either added or withdrawn as indicated. Data show cell lines 25 days after this change. X-axis shows DNA content (by FxCycle Far Red incorporation), while Y-axis shows EdU content. Equivalent data were observed 10 days after the changes in treatment (not shown). EdU negative control is the 3Crev cell line not pulsed by EdU, to allow definition of the EdU-positive cells. (**B**) Western blots showing latency-associated EBV gene expression in p16^INK4a^-null LCLs. Two p16^INK4a^-competent LCLs are shown for comparison. 3CHT-4HT cells are those established in the absence of 4HT.

Although outgrowth of EBNA3C-deficient lines (3CKO and 3CHT-never 4HT) was slower than EBNA3C-expressing ones, all the cell lines continued proliferating beyond 30 days – the time by which normal p16^INK4a^-competent B cells infected with EBNA3C-deficient viruses stopped proliferating and/or died (see above). By about 2.5 months cell lines were considered established and the culture conditions were reversed, ie 4HT was added to 3CHT-never 4HT cells, and withdrawn from 3CHT with 4HT cells. The removal/addition of 4HT did not consistently alter the proportion of cells synthesizing DNA at either 25 days (Figure 9A) or at 10 days (not shown). Western blotting of protein extracted from the cell lines as early after infection as was practical (about 1.5 months for most lines and about 3 months post-infection for 3CKO LCL) confirmed the EBNA3C status of the individual lines, and that the other EBV latency-associated proteins are generally expressed at similar levels in all the lines. The only exception is EBNA-LP, the expression of which is notoriously variable between LCLs (Figure 9B).

While we observed some slight differences in proliferation between cells expressing or lacking EBNA3C in the p16^INK4a^-null background, particularly early during the establishment of LCLs, here we have formally shown that the failure of EBNA3C-deficient EBVs to repress p16^INK4a^ expression is the central reason that these viruses normally fail to transform B cells into continuously proliferating cell lines.

## Discussion

Two EBV latency-associated nuclear proteins – EBNA3A and EBNA3C – cooperate to harness the polycomb system for the repression of two host tumour suppressor genes. *BCL2L11*, the first of these genes to be characterized, encodes BIM a pro-apoptotic BCL2-family member that lowers the apoptotic threshold of cells, particularly in cells of the immune system ([23], [24], [28]; reviewed in [51]). The second target gene, *INK4a*, encodes the cyclin dependent kinase inhibitor p16^INK4a^ that prevents the phosphorylation of the tumour suppressor Rb and the resulting hypophosphorylated Rb blocks entry of cells into S phase of the cell cycle ([25], [37]; reviewed in [1], [2]).

### Repressing p16^INK4a^ is essential for LCL outgrowth and proliferation

In this study we have explored further the role of p16^INK4a^ in B cell transformation and shown that its expression is the major barrier to the initial outgrowth and subsequent proliferation of LCLs produced by the infection of primary B cells with EBV. This was made possible using Leiden B cells carrying a homozygous genomic deletion that specifically ablates production of functional p16^INK4a^. These cells were infected with recombinant EBVs that express either a conditional EBNA3C or no EBNA3C. A comparison of p16-null LCLs with LCLs established from normal B cells showed unequivocally that, if p16^INK4a^ is not functional, then EBNA3C is unnecessary to sustain cell proliferation. Consistent with this, it was possible to transform p16-null B cells into LCLs with EBV, but without any functional EBNA3C ever having been expressed. A possible explanation for why EBV has evolved a mechanism for suppressing p16^INK4a^ expression became apparent from examining the outcome of attempted transformations of normal B cells with EBNA3C-deficient EBV. These experiments revealed that EBV infection induced p16^INK4a^ transcription in the first few days after infection – when EBNA2 transactivates directly (eg c-MYC) or indirectly (eg cyclin D2) inducers of cell cycle progression and hyperproliferation [49], [50]. It is likely that unscheduled entry into S-phase is interpreted as oncogene de-regulation, and activation of p16^INK4a^ transcription is a consequence. When the infecting virus expressed functional EBNA3C (and EBNA3A) there was a halt to the increase of p16^INK4a^ expression from about day 7 onwards. However, if functional EBNA3C was not expressed, transcription from *INK4a* continued uncontrolled and the level of mRNA progressively increased over the next 2–3 weeks, until finally most of the cells arrested and/or died. EBNA3A/3C-mediated inhibition of *INK4a* transcription is therefore a critical countermeasure for the virus to bypass an intrinsic host cell defense against oncogenic transformation. This ensures expansion of the infected B cell population and the initiation of long-term persistence (see below for further discussion). Strictly speaking, here EBNA3C and EBNA3A do not actually repress *INK4a* transcription, but rather block its activation. We assume this involves the recruitment of polycomb complexes to the *CDKN2A* locus, leading to H3K27me3 modifications on chromatin around the *INK4a* transcription start site as we see in established LCLs.

### Is *INK4a* the only target of EBNA3C and p16^INK4a^ the only barrier to overcome in B cell transformation?

Although the Leiden B cells fail to make functional p16^INK4a^, they do express the fusion protein p14/p16 that – because it includes the critical, conserved amino-terminal 25 amino acids of p14^ARF^ – has all the known functions of p14^ARF^ [35], [36]. Furthermore expression of p14/16 was elevated when EBNA3C is inactivated and there was a concomitant reduction of H3K27me3 at the *ARF* locus (data not shown). It was therefore surprising to discover that in the Leiden cells, increased p14/p16 expression had no apparent effect on proliferation or LCL outgrowth. This prompted us to investigate the pathway that is normally activated downstream of p14^ARF^. Since neither p53 nor p21^WAF1^ were activated and we could detect no impairment of p53 or p21^WAF1^ function, irrespective of the EBNA3C status of cells, we conclude that either the amount of p14/16 does not exceed a critical threshold sufficient to trigger the p53 pathway, or that in these EBV-infected B cells another factor – perhaps EBV-encoded – inhibits p14^ARF^ function. A good candidate for this is EBNA-LP that, in transient assays, can associate with and interfere with the capacity of p14^ARF^ to inhibit proliferation [52]. Genetic analysis of the role of EBNA-LP during the early stages of B cell transformation, when EBNA-LP is normally expressed at high levels, should help resolve this issue. A third possibility that cannot be excluded is that p14^ARF^ does not play a significant role in the response of any human cells to oncogenic stress – as has been reported for human fibroblasts and epithelial cells [1], [3].

Additional analysis of the Rb-E2F1 axis produced no evidence that EBNA3C compromises the action of these host proteins. Microarray analysis showed that in continuously proliferating B cells EBNA3C has no effect on the transcription of (n = 45) E2F1-target genes, with the exception of that encoding the tumour suppressor p73. The data suggest that EBNA3C expression is associated with very modest activation of *TP73* in cycling B cells. These data have not been validated at the protein level, but the changes in expression clearly have no significant effect on LCL proliferation. Furthermore, by growing LCLs to saturation density it was possible to show hypophosphorylation of Rb in the p16-null cells and that a p16^INK4a^-independent G1 arrest is unaffected by EBNA3C in the context of normal viral latency-associated gene expression. All the results are consistent with p16^INK4a^ being the major barrier to maintaining EBV-induced proliferation and the principal requirement of EBNA3C for LCL outgrowth is to restrain transcription of *INK4a*.

### p16^INK4a^ and the DNA damage response (DDR)

It was reported recently that EBNA3C helps attenuate a DDR that is particularly active in the first week after EBV infection of normal B cells *in vitro –* when the cells are beginning to cycle very rapidly [50]. It is striking that during the same period p16^INK4a^ appears to be actively transcribed. Although the increase in p16^INK4a^ is probably primarily associated with oncogene activation, one cannot exclude the possibility that the increase is partly a response to DNA damage. Such an increase in p16^INK4a^ has been described following treatment of several types of cell with various DNA-damaging stimuli (reviewed in [3]). One can also speculate that if the increase in p16^INK4a^ activates Rb, which then leads to the repression of E2F-regulated genes while c-MYC is constitutively active, then some cells might enter S phase with sub-optimal amounts of DNA precursors and/or replication enzymes and this could lead to stalled DNA replication that is ‘read’ as DNA damage and triggers phosphorylation of histone H2AX. So, although EBNA3C may have a direct effect on the ATM/CHK2 pathway that triggers the DDR as has been suggested [50], its absence could also exacerbate the response because of the accumulating p16^INK4a^. Indeed, when EBNA3C is inactivated by removal of 4HT in continuously cycling p16-competent LCLs, substantially more H2AX is phosphorylated than in p16-null LCLs treated in the same way – this strongly implicates p16^INK4a^ in the DDR of LCLs (Figure S7). It will require systematic genetic analysis of EBNA3C and its role during the first 2–3 weeks post-infection to disentangle the DDR and the p16^INK4a^ senescence response and their precise contributions to the inhibition of B cell transformation. Nevertheless, the data presented here clearly demonstrate that in the absence of active EBNA3C and p16^INK4a^, even if the DDR is not attenuated by EBNA3C, it is insufficient to prevent LCL outgrowth. In contrast, if p16^INK4a^ is present when EBNA3C is absent, proliferation and LCL outgrowth are completely blocked – suggesting a dominant role for p16^INK4a^ in the restriction of B cell transformation.

### Arrest vs. Death

When p16-competent cells were infected with EBNA3C-deficient EBVs, from about day 14 post-infection there was increasing evidence of viable cells arresting and escalating levels of cell death in the population. Since this increase in cell death does not appear to occur in the p16-null B cells infected with similar viruses, it suggests there may be some crosstalk between p16^INK4a^ and the apoptotic machinery. This would be consistent with the evidence that in lymphocytes the default pathway triggered by p16^INK4a^ is apoptosis rather than prolonged cell cycle arrest [53], [54]. However one must remember that EBNA3A and EBNA3C cooperate to repress not only *INK4a*, but also *BCL2L11*, encoding BIM. Since in the absence of EBNA3C there is a parallel increase in BIM RNA expression between days 7 and 15 (Figure S8), we suggest it is highly likely that in cells accumulating p16^INK4a^, the simultaneous increase in BIM will substantially reduce the threshold to apoptosis. Cells with high levels of BIM will die because of its capacity to bind and inactivate all anti-apoptotic BCL2-family members [51]. At this stage we cannot rule out the possibility of p14/p16 playing a role in the p53-independent induction of cell death in the p16-null background.

### Implications in EBV biology and cancer development

Most of the data on EBV persistence in humans are consistent with the viral genome residing long-term in a population of memory B cells (MBCs). It appears that to establish persistence EBV initially infects resting (naïve) B cells and drives these to proliferate as activated B-blasts [9], [55]. We assume that – by analogy to what occurs in culture – repression of *INK4a* is essential at this stage to ensure the transient proliferation of the infected population. This expanding population of activated B cells is then thought to either migrate into or initiate a germinal center, where the cells differentiate to centroblasts, centrocytes and finally MBCs. This process includes the regulated shutdown of latent EBV expression [55]. Since the repression of *INK4a* involves polycomb-mediated covalent histone modifications, it is possible the epigenetic memory of this will be passed to progeny cells and carried through into the MBC population, even if the initiators, EBNA3A and EBNA3C, are no longer expressed.

It is well established that polycomb-mediated gene repression is often a precursor to promoter DNA methylation (reviewed in [7], [56]), so it is reasonable to hypothesize that the B cells reprogrammed *in vivo* by EBV will be particularly prone to aberrant DNA methylation at the *CDKN2A* and *BCL2L11* loci during tumorigenesis. This is consistent with reports describing promoter methylation of these genes in EBV-positive B lymphomas and derived cell lines [24], [57]–[59]

In summary, we have formally demonstrated that the epigenetic repression of p16^INK4a^ expression by EBV is central to the virus's ability to infect resting B cells and establish latency – and probably persist life-long *in vivo*. By targeting *INK4a* (and *BCL2L11*) EBV has evolved an effective countermeasure to oncogenic stress triggered by the early stages of infection. To our knowledge this particular strategy is unique among tumour viruses. Finally by making use of Leiden p16-null B cells, it has been possible to generate LCLs in which multiple EBV-regulated polycomb-targeted genes – including *CDKN2A* – can be switched on and off without altering the rate of cell proliferation or stage of cell differentiation.

## Materials and Methods

### Ethics statement

This study was conducted according to the principles expressed in the Declaration of Helsinki. Patient peripheral blood leukocyte (PBL) samples were obtained from archival stocks of Leiden University Medical Center. These were collected as part of an ongoing international collaborative study of CDKN2A mutations and malignant melanoma (project RUL 99-1932). Written, informed consent was obtained from participants at the time of collection.

### Cell isolation and culture

LCL under continuous culture were grown in RPMI-1640 medium supplemented with Penicillin/Streptomycin and L-Glutamine and 10% FCS. For initial outgrowth of LCLs and infection experiments, peripheral blood leukocytes (PBLs) were isolated from buffy-coat residues (UK blood transfusion service) by centrifugation over ficoll. Where indicated, B cells were further purified from PBLs by binding to CD19 microbeads (Miltenyi Biotec) and magnetically separating using positive selection on an autoMACS separator (Miltenyi Biotec). Isolated cells were kept at 1–2×10^6^ cells/ml in RPMI-1640 supplemented with L-glutamine, 500 ng/ml Cyclosporine A (Sigma) and 15% FCS (which we had batch-tested for LCL outgrowth efficiency – PAA Laboratories) in a 37°C incubator (5% CO_2_) until infection (which was within 24 hours of isolation). Gamma-irradiation of cells was performed as described previously [60].

### Infection of B cells with recombinant EBVs

The recombinant viruses used in this study were wild-type B95-8 BAC [61], EBNA3C knockout and revertants [23] and the EBNA3C-estrogen receptor fusion 3CHT [25]. Infection and outgrowth of LCLs was generally as described previously ([25], [26]. For cells to be analysed in the first month post-infection, PBLs were seeded at 2×10^6^ cells per well in 24 well plates and infected with 1.2–1.5×10^5^ Raji infectious units of virus. Medium was refreshed (approximately 60% of medium removed and replaced with fresh) after 24 hours and twice weekly thereafter. Cyclosporine A was retained in the medium for the first two weeks of outgrowth. As cell cultures saturated, approximately 75% of the culture was discarded for each split. CD19-purified B cells (mixed from four donors) were seeded for infection in 5 ml at 2×10^6^ cells/ml in 25 cm^2^ flasks stood upright. Approximately 5×10^5^ infectious units of virus (1.5×10^5^ units for 3CHT virus infections) were added to the cultures and medium was changed as for PBLs. These cell cultures were expanded to larger volumes and flasks as their numbers increased.

For the first infection of Leiden PBLs, cells were recovered from liquid nitrogen and 10^6^ cells per well were seeded in a 24 well plate. Four infections (two with each of two independently generated 3CHT recombinant EBVs – [25] were performed with 100 µl of virus supernatant (approximately 10^4^ infectious particles). The four cell lines were grown out in the presence of 4HT. The second Leiden PBL infection was hampered by the very low viability of the cells after thawing, resulting in fewer infected cells and longer times to establish the cell lines. Separate infections were undertaken with 3CKO and 3Crev and with 3CHT virus either in the presence or absence 4HT (using approximately 5×10^4^ infectious particles per well).

### Cell proliferation assays

Cell proliferation was assessed by measuring the incorporation into DNA of nucleotide analogues (BrdU or EdU). BrdU incorporation was assessed as described previously [25]. EdU (Life Technologies) incorporation was assessed as follows. For time courses of newly infected cells, infected cells were cultured in 1.5 ml in wells of a 24 well plate. Five hundred microlitres of this culture (or 300 µl where cells were dense enough to change the colour of the medium) was transferred to a new well in a final volume of 1 ml. After 24 hours, EdU (15 µM) was added in a volume of 500 µl to give a final concentration of 5 µM. Cells were harvested after a sixteen hours in the presence of EdU. For established cell lines, cells were seeded at 3×10^5^ cells/ml in 4 ml in a well of a 6 well plates. After 20 hours, culture was supplemented with 1 ml of warm medium containing EdU for a final concentration of 10 µM. Cells were harvested after 1 hour in the presence of EdU. Cells were washed once in cold PBS, then re-suspended in 500 µl PBS containing Violet Fixable Dead Cell Stain (Life Technologies) at 1 µl/ml. Cells were kept on ice in the dark for 30 minutes, then washed once with PBS, once with PBS/1% BSA, re-suspended in 100 µl PBS, and fixed by addition of 400 µl 100% ethanol. After >1 hour on ice, cells were rehydrated in 500 µl PBS/BSA for 15 minutes, then pelleted and re-suspended in 100 µl PBS/BSA. EdU was labeled by Click chemistry using an azide-derivative of AF488 dye according to the manufacturer's instructions (Life Technologies), before washing cells twice with PBS/BSA and re-suspending in 500 µl PBS/BSA supplemented with either DRAQ5 (Biostatus Ltd - 2 µl/ml) or FxCycle Far Red (1 µl/ml) to stain for DNA content.

Cell fluorescence was measured on either an LSR II (Becton Dickinson) or iCyt ec800 (Sony) flow cytometer. Single cells were gated based on Propdium Iodide/DRAQ5/FxCycle Far Red fluorescence (comparing fluorescence area to width or height at 633/690). Fluorescence measured by 405/450 filters indicated live/dead status, and only live cells were included for assessing proliferation by EdU (488/530).

### Western blotting

Western blotting was performed as described previously [23]. Anti-EBV antibodies used are as described previously [26] with the addition of the anti-EBNA-LP antibody clone 4D3 [62]. Antibodies and western blotting for p53, p53-phospho-ser20, p21^WAF1^, Rb, phospho-Rb and p107 are as described in [25], [60]. The EZH2 antibody used was from Active Motif (39934), anti-p16^INK4a^ JC8 was a gift from Gordon Peters (CRUK), anti-p16^INK4a^ DCS50.1 from Abcam. Throughout gamma-tubulin was probed with mAB GTU-88 (Sigma).

### Quantitative reverse transcript (qRT)-PCR

For qRT-PCR RNA was isolated using RNeasy mini kit (Qiagen) with DNase digestion according to the manufacturer's protocol. RNA was reverse transcribed into cDNA using the SuperScript III First-Strand Synthesis Supermix for qRT-PCR (Life Technologies), using a 55°C incubation to facilitate melting of the GC-rich first exon of p16^INK4a^ transcripts. Quantitive (q)PCR was generally undertaken on the cDNA and analysed as described previously [25], using platinum SYBR green qPCR SuperMix UDG kit (Invitrogen) for SYBR green-based qPCR assays, and using FAST BLUE qPCR MasterMix Plus dTTP (Eurogentec) for Taqman probe-based assays. Primer sets for the exon 1 *INK4a* assay and the *RPLP0*, *GNB2L1* and *ALAS1* endogenous control genes are described in [25]. Total *CDKN2A* transcript quantity was measured using the Hs00923894_m1 Taqman assay (Life Technologies) spanning the junction between exons 2 and 3. *BIM* (*BCL2L11*) assay is as described in [28]. Primers used for p21^WAF1^ were: p21-fwd ctggagactctcagggtcgaa and p21-rev gcggattagggcttcctctt.

### Chromatin immunoprecipations

ChIP assays and qPCR analysis were performed essentially as described previously: H3K27me3 and H3K4me3 [25] and EBNA3C-TAP [28]. The primer pairs for the transcriptional start sites for p15^INK4b^ and p14^ARF^ are described in [63].

### Microarray analysis

To generate a balanced set of RNA samples either expressing or lacking functional EBNA3C, cells were cultured and RNA extracted as described below and illustrated in Figure S2: 4HT was washed out of cell lines that had been established in the presence of 4HT (day zero). These cells were cultured in the absence of 4HT, alongside the same cell line maintained in the presence of 4HT. After thirty-two days, the cells growing in the presence of 4HT were again split into two and one culture washed and subsequently grown in the absence of 4HT. Those cells grown in the absence of 4HT were also split into two cultures. To one 4HT was re-added. At day 36, cells were seeded at 3×10^5^ cells/ml and RNA harvested the following day, producing samples whose conditions had been altered either 5 or 37 days previously (Figure S2). The cultures whose conditions had been changed on day 32 were grown a further 28 days (ie 33 days total culture in their new conditions) and RNA was isolated as above. Thus, each cell line yielded six samples: two from cells grown in the absence of 4HT for 33 or 37 days, one always grown in the presence of 4HT, one grown with 4HT for 33 days after a period of 32 days without HT, and samples five days after 4HT withdrawal or re-addition.

RNA was reverse transcribed and amplified (using the Applause WT-Amp Plus ST kit – NuGen) and hybridized to Affymetrix Human Exon 1.0ST microarrays by UCL Genomics. Gene level analysis was performed using the MMBGX algorithm [64] to generate gene-level data according ENSEMBL genome annotation version 64, as mapped to the Exon microarray by AnnMap (formerly X:Map), broadly as described previously [65], [66].

Statistical analysis of microarray data was performed using Partek Genomic Suite v6.5 (Partek Inc). The differential expression of genes was assessed by a 3-way ANOVA model using Method of Moments [67]. The model accounts for the cell line of origin (C), and the factorial combination of whether the cells are grown with or without 4HT (H) and the time of treatment (T) - classed as 5 days or >30 days - according to the formula: Yijkl = μ+Ci+H*Tjk+εijkl where Yijkl represents the lth observation on the ith cell line jth 4HT status kth Treatment term; μ is the common effect for the whole experiment. εijkl represents the random error present in the lth observation on the ith cell line ID jth HT status kth Treatment term. The errors εijkl are assumed to be normally and independently distributed with mean 0 and standard deviation δ for all measurements. Samples having different 4HT treatments for >30 days were compared using the contrast method (Partek Genomics Suite). For visualisation, variations in gene expression between cell lines were removed using the Remove Batch Effect tool in Partek Genomics Suite, based on the same ANOVA model used to identify the differentially regulated genes.

## Supporting Information

Figure S1 **EBNA3C-TAP directly targeted to sites across the** ***INK4b-ARF-INK4a*** **locus.** (**A**) LCLs were established by infection with a previously characterized virus that expresses a tagged version of EBNA3C, which includes the FLAG epitope [28]. Anti-FLAG antibody was used to perform ChIP. ChIP using cells infected with a wild type virus expressing non-tagged EBNA3C was also performed to show antibody specificity. The histogram bars represent the ratio of chromatin precipitated with the antibody relative to input, as judged by qPCR using the primer pairs indicated. Primer pairs amplifying close to the p15^INK4b^ and p14^ARF^ TSS were described previously [63]. Primer pairs that amplify close to the TSS of *MCM6* or *BIM* and *RASGRP1* genes were used as negative or positive controls, respectively, of previously characterized EBNA3C target sites [28]. Other primers are as described in main article. The error bars represent the standard deviation from triplicate PCR assays. These data are representative of five ChIP experiments in two different LCL backgrounds. B. Schematic of the *INK4b-ARF-INK4a* locus showing the sites where EBNA3C-TAP was detected.
(PDF)

Figure S2 **Schematic representation of microarray strategy.** Horizontal lines represent ongoing growth either in the presence (red lines) or absence (blue lines) of 4HT. Lines start at the time point (indicated at top) at which the culture conditions were initially changed. Block arrows indicate the time points at which RNA was harvested for Microarray analysis, their color representing 4HT status as shown. The experiment was initiated with cells that had been recovered from aliquots stored approximately 3 months post-infection.
(PDF)

Figure S3 **Microarray analysis of** ***CDKN2A*** **gene regulation by EBNA3C in p16-null LCLs.** This dot-plot indicates the gene level expression values measured by the exon microarray for cell lines after >30 days of treatment. Vertical axis shows a log2 scale for gene expression (ie each integer represents a doubling of gene expression). X-axis separates samples from cells grown in the presence of 4HT (red) from those in the absence of 4HT (blue). Shape of each data point indicates the cell line of origin of each sample, as indicated in the key. Note that some sort of clonal selection process appears to have resulted in one cell line (A2) losing the ability to repress *CDKN2A*.
(PDF)

Figure S4 **Hypophosphorylated Rb and G1-arrest in a p16-null LCL.** (**A**) Western blots showing the phosphorylation status of Rb (hypo- the faster migrating bands and hyper- the slower migrating bands indicated). Total protein was extracted from two p16-null LCLs that were either harvested 24 hours after feeding (Cyc), left for 120 hours to reach saturation density (Sat 120 h) or left 96 hours, re-fed and harvested 24 hours later (Cyc*). (**B**) Samples of cells from LCL 3CHT-C2, fixed, stained with PI and analysed by flow cytometry.
(PDF)

Figure S5 **EdU incorporation in primary B cells infected with EBNA3C competent and deficient viruses.** FACS plots showing the proportion of live cells incorporating EdU into their DNA during a 16 hour-pulse, either 7 or 20 days post infection. EdU measurement is shown on the vertical axis, while the horizontal axis shows DRAQ5, which is proportional to the cell's DNA content. EdU-positive cells (as identified by comparison to proliferating 3Crev-infected cells not pulsed with EdU) are those within the pink box, whose percentage is shown for each plot. Note also the paucity of live cells in 3C-deficient cell lines at 20 days post infection. By 27 days, there were not sufficient live cells to reliably assess cell proliferation by EdU incorporation.
(PDF)

Figure S6 **Cell proliferation and viability after primary infection of B cells with recombinant EBVs: PBLs from donor D13.** Data are presented essentially as described for Figure 8C and D. Ficoll-purified lymphocytes, from a buffy-coat residue (donor D13), were infected by the viruses indicated. The proportion of proliferating cells (**A**) was assessed by EdU incorporation over 16 hours. Cell viability was assessed by Live/Dead staining (**B**). The sampling time after infection, and the virus used to infect the cells are as indicated.
(PDF)

Figure S7 **DNA damage in p16-competent LCL 3CHT cells.** Western blot of phosphorylated H2AX (an indicator of DNA damage – also known as gamma-H2AX) in two p16-null LCL 3CHT lines (corresponding to A2 and C2 in Figure 1) and two competent LCL 3CHT lines (-A and -C) cultured for 21 days with (+) or without (−) 4HT. Gamma-tubulin was used as a loading control. A significant increase in phosphorylation of H2AX was detected only in p16-competent lines cultured without 4HT. Data are representative of two independent experiments each including at least two p16-null LCL 3CHT lines.
(PDF)

Figure S8 **Changes in** ***BIM*** **(** ***BCL2L11*** **) gene expression after EBV infection of primary B cells.** Expression of *BIM* RNA as measured by qPCR after infection with wild-type/revertant EBV-BACs, as compared to 3CKO EBV. The orange line represents the average expression (and standard deviation) of independent infections with four different wild-type or revertant EBVs. This is compared to infections with 3CKO (blue) and 3CHT in the absence of 4HT (purple) and with 3CHT+4HT (red), where the error bars indicate standard deviation of triplicate qPCRs. Expression data were normalized to expression of *ALAS1*, *GNB2L1* and *RPLP0* and expression values are expressed relative to the average of all data points. Note the higher *BIM* expression in the EBNA3C-deficient infections, in keeping with EBNA3C's role as a repressor of BIM transcription. Also, as seen for *p16^INK4a^* expression (Figure 7), there is a slightly reduced efficiency of repression of *BIM* by the 3CHT virus grown with 4HT as compared to wild-type viruses. The drop in *BIM* RNA levels after two weeks probably occur because cells with higher *BIM* RNA levels die as a critical threshold of BIM protein is passed.
(PDF)

Table S1 **Genes regulated by the inactivation of EBNA3C.**
(XLSX)
