# Correction: Induction of p16^INK4a^ Is the Major Barrier to Proliferation when Epstein-Barr Virus (EBV) Transforms Primary B Cells into Lymphoblastoid Cell Lines

**DOI:** 10.1371/annotation/2c4b89c1-6d4a-4bf3-9467-9367227d7e74

**Published:** 2013-03-05

**Authors:** Lenka Skalska, Robert E. White, Gillian A. Parker, Ernest Turro, Alison J. Sinclair, Kostas Paschos, Martin J. Allday

Dr. Ernest Turro was not included in the author byline. He should be listed as the fourth author and is affiliated with Department of Oncology, University of Cambridge, Hills Road, Cambridge CB2 0XZ, United Kingdom and Cancer Research UK, Cambridge Research Institute, Robinson Way, Cambridge CB2 0RE, United Kingdom. The contribution of this author is as follows: contributed analysis tools and analyzed data.

The correct citation is: Skalska L, White RE, Parker GA, Turro E, Sinclair AJ, et al. (2013) Induction of p16INK4a Is the Major Barrier to Proliferation when Epstein-Barr Virus (EBV) Transforms Primary B Cells into Lymphoblastoid Cell Lines. PLoS Pathog 9(2): e1003187. doi:10.1371/journal.ppat.1003187

