# Supplementary material for: Induction of p16INK4a Is the Major Barrier to Proliferation when Epstein-Barr Virus (EBV) Transforms Primary B Cells into Lymphoblastoid Cell Lines
Source: PLoS Pathog. 2013 Feb 21;9(2):e1003187. doi: 10.1371/journal.ppat.1003187 (PMC3578823; doi:10.1371/journal.ppat.1003187)
Supplement: Figure S1 — EBNA3C-TAP directly targeted to sites across the INK4b-ARF-INK4a locus. (A) LCLs were established by infection with a previously characterized virus that expresses a tagged version of EBNA3C, which includes the FLAG epitope [28]. Anti-FLAG antibody was used to perform ChIP. ChIP using cells infected with a wild type virus expressing non-tagged EBNA3C was also performed to show antibody specificity. The histogram bars represent the ratio of chromatin precipitated with the antibody relative to input, as judged by qPCR using the primer pairs indicated. Primer pairs amplifying close to the p15INK4b and p14ARF TSS were described previously [63]. Primer pairs that amplify close to the TSS of MCM6 or BIM and RASGRP1 genes were used as negative or positive controls, respectively, of previously characterized EBNA3C target sites [28]. Other primers are as described in main article. The error bars represent the standard deviation from triplicate PCR assays. These data are representative of five ChIP experiments in two different LCL backgrounds. B. Schematic of the INK4b-ARF-INK4a locus showing the sites where EBNA3C-TAP was detected. (PDF) [file ppat.1003187.s001.pdf]

A

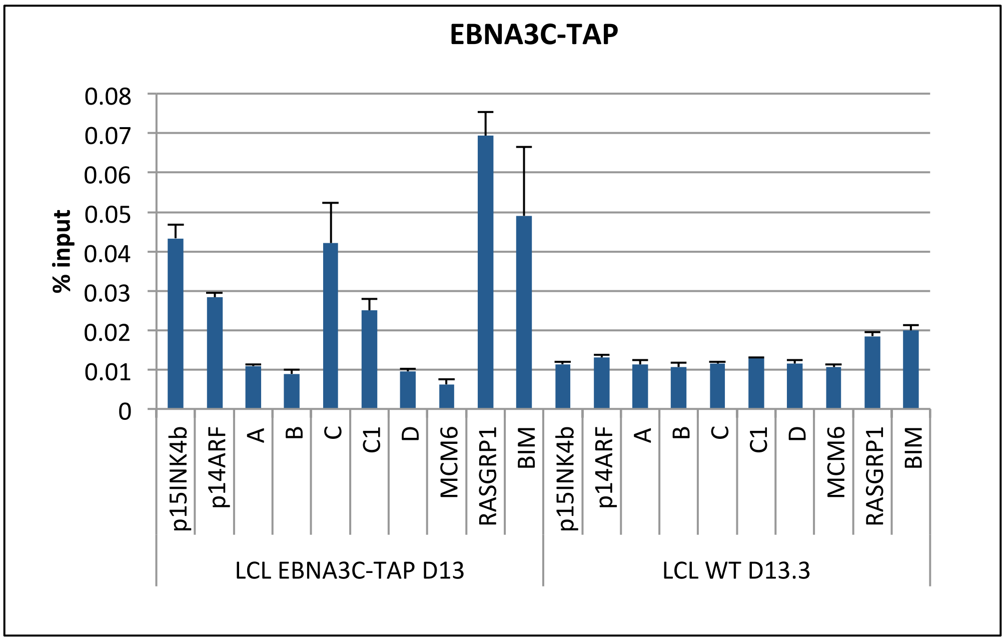

B

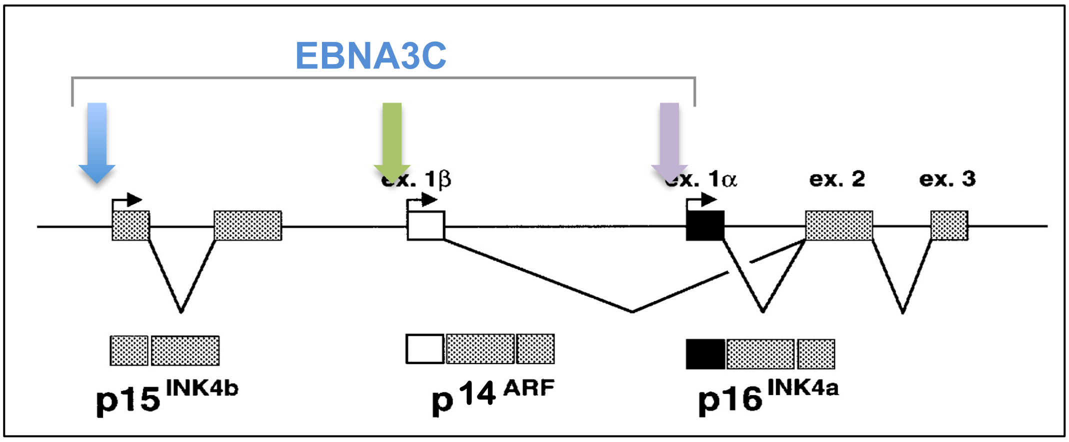

Figure S1. EBNA3C-TAP directly targeted to sites across the *INK4b-ARF-INK4a* locus. **A.** LCLs were established by infection with a previously characterized virus that expresses a tagged version of EBNA3C, which includes the FLAG tag [28]. Anti-FLAG antibody was used to perform ChIP. ChIP using cells infected with a wild type virus expressing non-tagged EBNA3C was also performed to show antibody specificity. The histogram bars represent the ratio of chromatin precipitated with the antibody relative to input, as judged by qPCR using the primer pairs indicated. Primer pairs amplifying close to the $p15^{INK4b}$ and $p14^{ARF}$ TSS were described previously [63]. Primer pairs that amplify close to the TSS of *MCM6* or *BIM* and *RASGRP1* genes were used as negative or positive controls, respectively, of previously characterized EBNA3C target sites [28]. Other primers are as described in main article. The error bars represent the standard deviation from triplicate PCR assays. These data are representative of five ChIP experiments in two different LCL backgrounds**. B.** Schematic of the ***INK4b-ARF-INK4a*** locus showing the sites where EBNA3C-TAP was detected.
