# Supplementary material for: Induction of p16INK4a Is the Major Barrier to Proliferation when Epstein-Barr Virus (EBV) Transforms Primary B Cells into Lymphoblastoid Cell Lines
Source: PLoS Pathog. 2013 Feb 21;9(2):e1003187. doi: 10.1371/journal.ppat.1003187 (PMC3578823; doi:10.1371/journal.ppat.1003187)
Supplement: Figure S2 — Schematic representation of microarray strategy. Horizontal lines represent ongoing growth either in the presence (red lines) or absence (blue lines) of 4HT. Lines start at the time point (indicated at top) at which the culture conditions were initially changed. Block arrows indicate the time points at which RNA was harvested for Microarray analysis, their color representing 4HT status as shown. The experiment was initiated with cells that had been recovered from aliquots stored approximately 3 months post-infection. (PDF) [file ppat.1003187.s002.pdf]

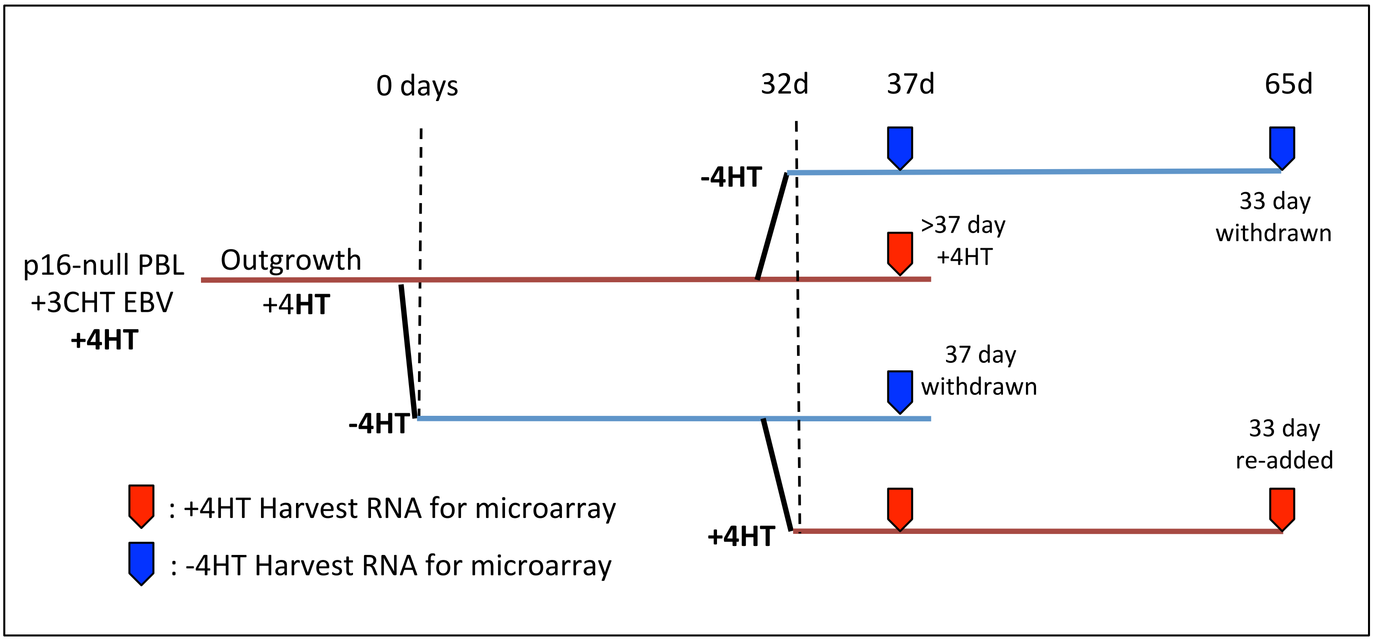

Figure S2. Schematic representation of microarray strategy. Horizontal lines represent ongoing growth either in the presence (red lines) or absence (blue lines) of 4HT. Lines start at the time point (indicated at top) at which the culture conditions were initially changed. Block arrows indicates the time points at which RNA was harvested for Microarray analysis, their colour representing 4HT status as indicated. The experiment was initiated with cells that had been recovered from aliquots stored approximately 3 months post-infection.
