# Supplementary material for: Induction of p16INK4a Is the Major Barrier to Proliferation when Epstein-Barr Virus (EBV) Transforms Primary B Cells into Lymphoblastoid Cell Lines
Source: PLoS Pathog. 2013 Feb 21;9(2):e1003187. doi: 10.1371/journal.ppat.1003187 (PMC3578823; doi:10.1371/journal.ppat.1003187)
Supplement: Figure S3 — Microarray analysis of CDKN2A gene regulation by EBNA3C in p16-null LCLs. This dot-plot indicates the gene level expression values measured by the exon microarray for cell lines after >30 days of treatment. Vertical axis shows a log2 scale for gene expression (ie each integer represents a doubling of gene expression). X-axis separates samples from cells grown in the presence of 4HT (red) from those in the absence of 4HT (blue). Shape of each data point indicates the cell line of origin of each sample, as indicated in the key. Note that some sort of clonal selection process appears to have resulted in one cell line (A2) losing the ability to repress CDKN2A. (PDF) [file ppat.1003187.s003.pdf]

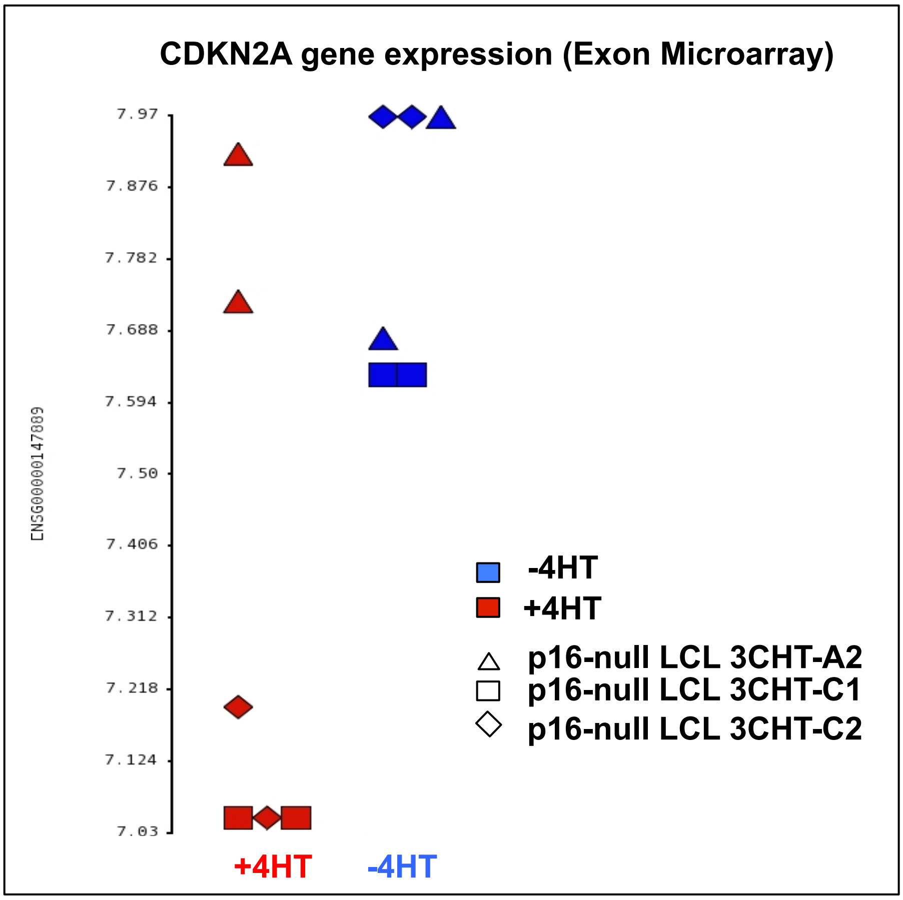

Figure S3. Microarray analysis of *CDKN2A* gene regulation by EBNA3C in p16^{null} LCLs. This dot-plot indicates the gene level expression values measured by the exon microarray for cell lines after >30 days of treatment. Vertical axis shows a log2 scale for gene expression (ie each integer represents a doubling of gene expression). X-axis separates samples from cells grown in the presence of 4HT (red) from those in the absence of 4HT (blue). Shape of each data point indicates the cell line of origin of each sample, as indicated in the key. Note that some sort of clonal selection process has resulted in one cell line (A2) having lost the ability to repress *CDKN2A*.
