# Supplementary material for: Induction of p16INK4a Is the Major Barrier to Proliferation when Epstein-Barr Virus (EBV) Transforms Primary B Cells into Lymphoblastoid Cell Lines
Source: PLoS Pathog. 2013 Feb 21;9(2):e1003187. doi: 10.1371/journal.ppat.1003187 (PMC3578823; doi:10.1371/journal.ppat.1003187)
Supplement: Figure S4 — Hypophosphorylated Rb and G1-arrest in a p16-null LCL. (A) Western blots showing the phosphorylation status of Rb (hypo- the faster migrating bands and hyper- the slower migrating bands indicated). Total protein was extracted from two p16-null LCLs that were either harvested 24 hours after feeding (Cyc), left for 120 hours to reach saturation density (Sat 120 h) or left 96 hours, re-fed and harvested 24 hours later (Cyc*). (B) Samples of cells from LCL 3CHT-C2, fixed, stained with PI and analysed by flow cytometry. (PDF) [file ppat.1003187.s004.pdf]

A

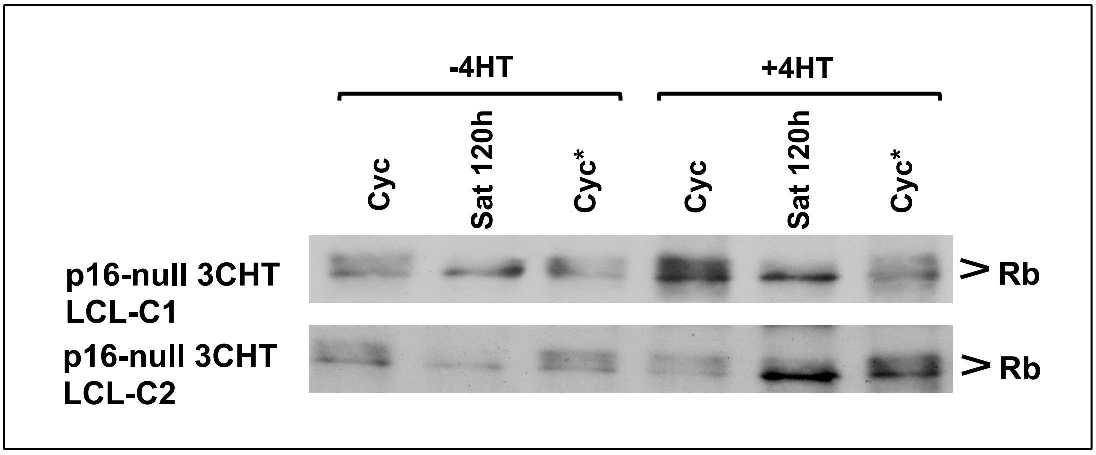

B

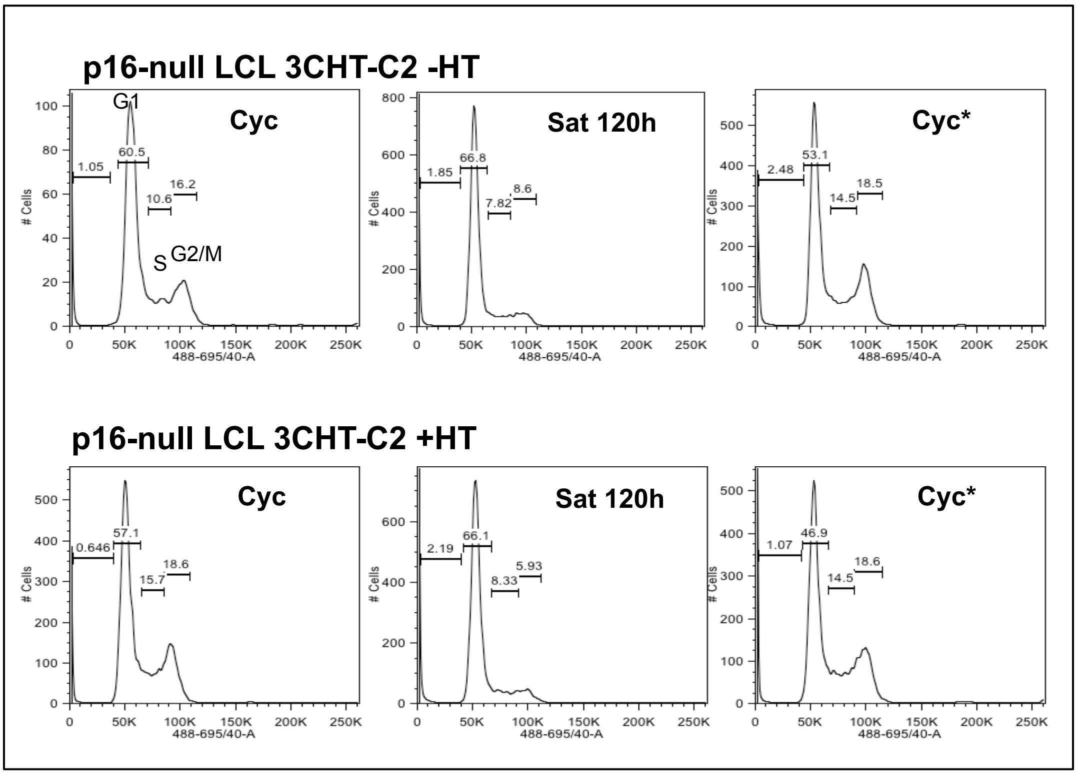

Figure S4. Hypophosphorylated Rb and G1-arrest in a p16-null LCL. (A) Western blots showing the phosphorylation status of Rb (hypo- the faster migrating bands and hyper- the slower migrating bands indicated). Total protein was extracted from two p16-null LCLs that were either harvested 24 hours after feeding (Cyc), left for 120 hours to reach saturation density (Sat 120h) or left 96 hours, re-fed and harvested 24 hours later (Cyc*). (B) Samples of cells from LCL 3CHT-C2, fixed, stained with PI and analysed by flow cytometry.
