# Supplementary material for: Induction of p16INK4a Is the Major Barrier to Proliferation when Epstein-Barr Virus (EBV) Transforms Primary B Cells into Lymphoblastoid Cell Lines
Source: PLoS Pathog. 2013 Feb 21;9(2):e1003187. doi: 10.1371/journal.ppat.1003187 (PMC3578823; doi:10.1371/journal.ppat.1003187)
Supplement: Figure S5 — EdU incorporation in primary B cells infected with EBNA3C competent and deficient viruses. FACS plots showing the proportion of live cells incorporating EdU into their DNA during a 16 hour-pulse, either 7 or 20 days post infection. EdU measurement is shown on the vertical axis, while the horizontal axis shows DRAQ5, which is proportional to the cell's DNA content. EdU-positive cells (as identified by comparison to proliferating 3Crev-infected cells not pulsed with EdU) are those within the pink box, whose percentage is shown for each plot. Note also the paucity of live cells in 3C-deficient cell lines at 20 days post infection. By 27 days, there were not sufficient live cells to reliably assess cell proliferation by EdU incorporation. (PDF) [file ppat.1003187.s005.pdf]

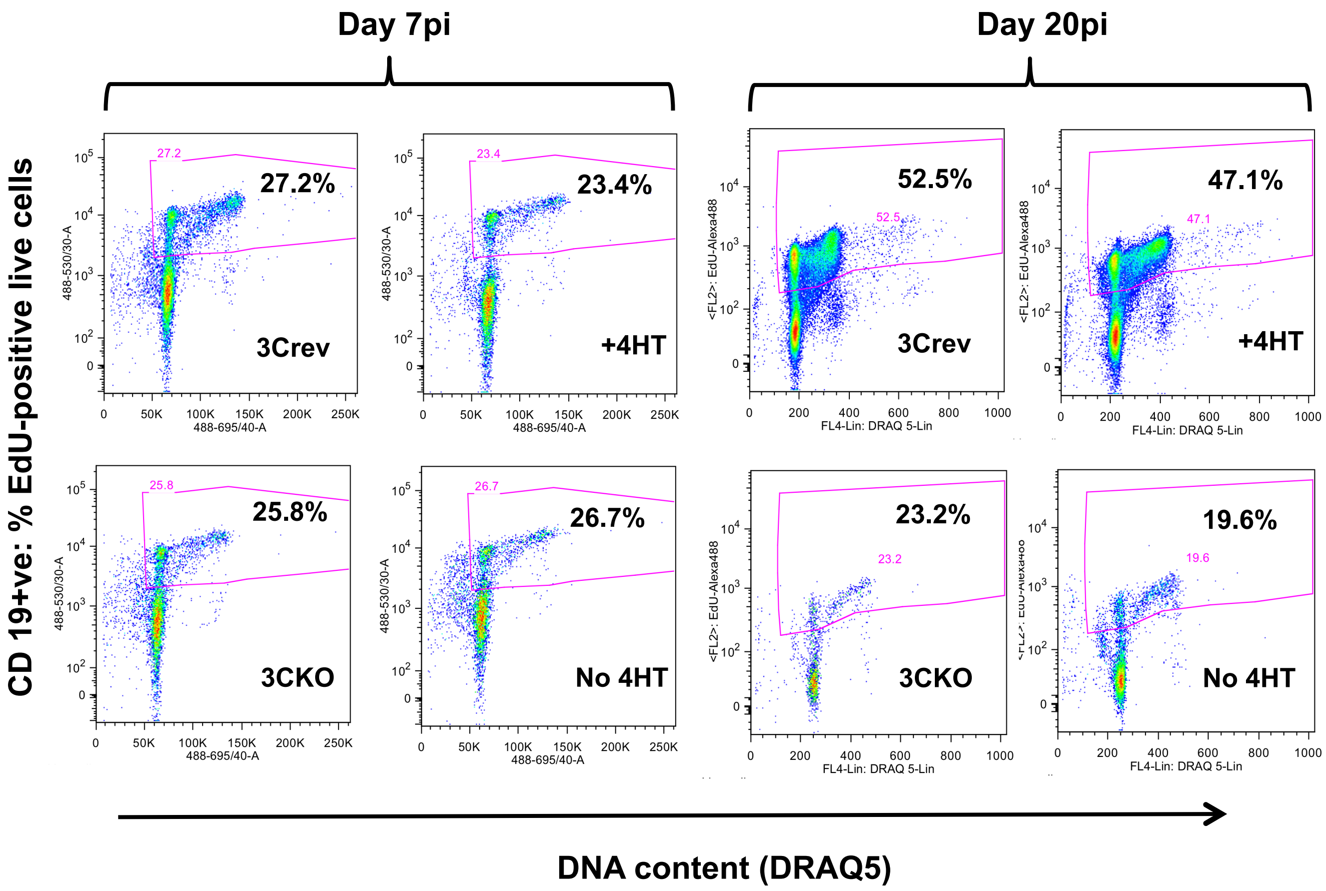

Figure S5. EdU incorporation in primary B cells infected with EBNA3C competent and deficient viruses. FACS plots showing the proportion of live cells incorporating EdU into their DNA during a 16 hour pulse, either 7 or 20 days post infection. EdU measurement is shown on the vertical axis, while the horizontal axis shows DRAQ5, which is proportional to the cell's DNA content. EdU-positive cells (as identified by comparison to proliferating 3Crev-infected cells not pulsed with EdU) are those within the pink box, whose percentage is shown for each plot. Note also the paucity of live cells in 3C-deficient cell lines at 20 days post infection. By 27 days, there were not sufficient live cells to reliably assess cell proliferation by EdU incorporation.
