# Supplementary material for: Induction of p16INK4a Is the Major Barrier to Proliferation when Epstein-Barr Virus (EBV) Transforms Primary B Cells into Lymphoblastoid Cell Lines
Source: PLoS Pathog. 2013 Feb 21;9(2):e1003187. doi: 10.1371/journal.ppat.1003187 (PMC3578823; doi:10.1371/journal.ppat.1003187)
Supplement: Figure S6 — Cell proliferation and viability after primary infection of B cells with recombinant EBVs: PBLs from donor D13. Data are presented essentially as described for Figure 8C and D. Ficoll-purified lymphocytes, from a buffy-coat residue (donor D13), were infected by the viruses indicated. The proportion of proliferating cells (A) was assessed by EdU incorporation over 16 hours. Cell viability was assessed by Live/Dead staining (B). The sampling time after infection, and the virus used to infect the cells are as indicated. (PDF) [file ppat.1003187.s006.pdf]

**A**

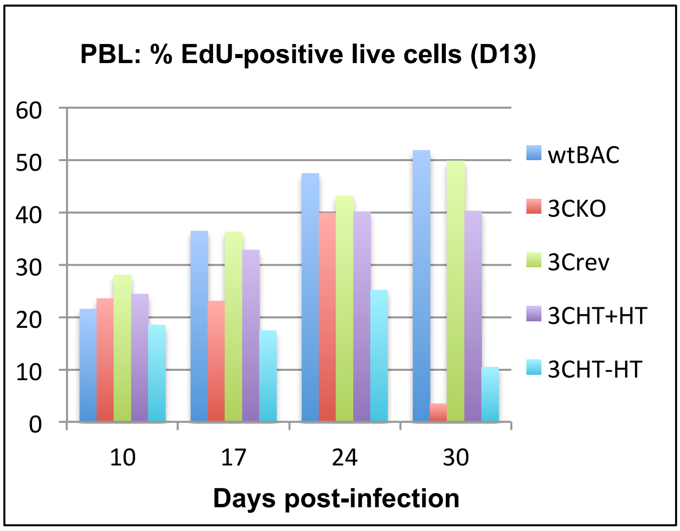


**B**

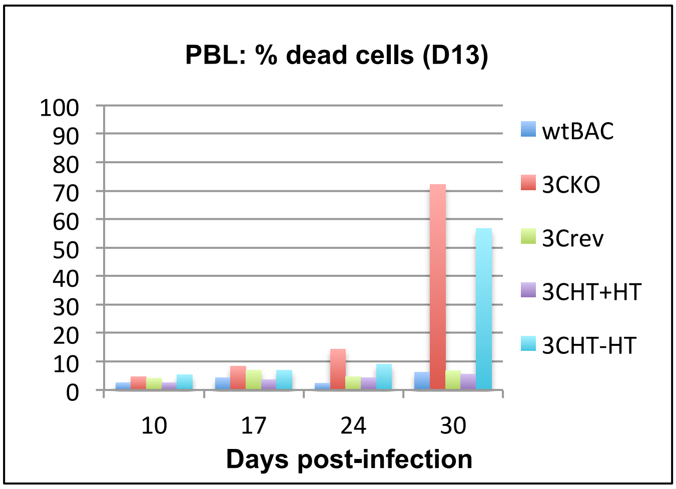


**Figure S6. Cell proliferation and viability after primary infection of B cells with by recombinant EBVs: PBLs from donor D13**. Data are presented essentially as described for figure 8C and D: Ficoll-purified bulk lymphocytes from a buffy coat residue (from donor D13) were infected by the viruses indicated, at day 0. The proportion of proliferating cells (**A**) was assessed by EdU incorporation over 16 hours. Cell viability was assessed by Live/Dead staining (**B**). The sampling time after infection, and the virus used to infect the cells are as indicated.
