# Supplementary material for: Induction of p16INK4a Is the Major Barrier to Proliferation when Epstein-Barr Virus (EBV) Transforms Primary B Cells into Lymphoblastoid Cell Lines
Source: PLoS Pathog. 2013 Feb 21;9(2):e1003187. doi: 10.1371/journal.ppat.1003187 (PMC3578823; doi:10.1371/journal.ppat.1003187)
Supplement: Figure S7 — DNA damage in p16-competent LCL 3CHT cells. Western blot of phosphorylated H2AX (an indicator of DNA damage – also known as gamma-H2AX) in two p16-null LCL 3CHT lines (corresponding to A2 and C2 in Figure 1) and two competent LCL 3CHT lines (-A and -C) cultured for 21 days with (+) or without (−) 4HT. Gamma-tubulin was used as a loading control. A significant increase in phosphorylation of H2AX was detected only in p16-competent lines cultured without 4HT. Data are representative of two independent experiments each including at least two p16-null LCL 3CHT lines. (PDF) [file ppat.1003187.s007.pdf]

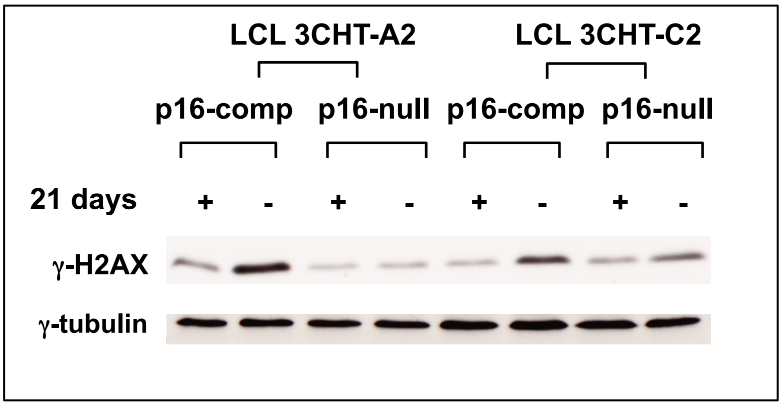

Figure S7. DNA damage in p16-competent LCL 3CHT. Western blot of phosphorylated (gamma)-H2AX (an indicator of DNA damage) in two p16-null LCL 3CHT (corresponding to A2 and C2 in Figure 1) and two competent LCL 3CHT (-A and -C) cultured for 21 days with (+) or without (-) 4HT. Gamma-tubulin was used as a loading control. A significant increase in phosphorylation of H2AX (gamma-H2AX) was detected only in p16-competent lines cultured without 4HT. Data are representative of two independent experiments each including at least two p16-null LCL 3CHT lines.
