# Supplementary material for: Induction of p16INK4a Is the Major Barrier to Proliferation when Epstein-Barr Virus (EBV) Transforms Primary B Cells into Lymphoblastoid Cell Lines
Source: PLoS Pathog. 2013 Feb 21;9(2):e1003187. doi: 10.1371/journal.ppat.1003187 (PMC3578823; doi:10.1371/journal.ppat.1003187)
Supplement: Figure S8 — Changes in BIM ( BCL2L11 ) gene expression after EBV infection of primary B cells. Expression of BIM RNA as measured by qPCR after infection with wild-type/revertant EBV-BACs, as compared to 3CKO EBV. The orange line represents the average expression (and standard deviation) of independent infections with four different wild-type or revertant EBVs. This is compared to infections with 3CKO (blue) and 3CHT in the absence of 4HT (purple) and with 3CHT+4HT (red), where the error bars indicate standard deviation of triplicate qPCRs. Expression data were normalized to expression of ALAS1, GNB2L1 and RPLP0 and expression values are expressed relative to the average of all data points. Note the higher BIM expression in the EBNA3C-deficient infections, in keeping with EBNA3C's role as a repressor of BIM transcription. Also, as seen for p16INK4a expression (Figure 7), there is a slightly reduced efficiency of repression of BIM by the 3CHT virus grown with 4HT as compared to wild-type viruses. The drop in BIM RNA levels after two weeks probably occur because cells with higher BIM RNA levels die as a critical threshold of BIM protein is passed. (PDF) [file ppat.1003187.s008.pdf]

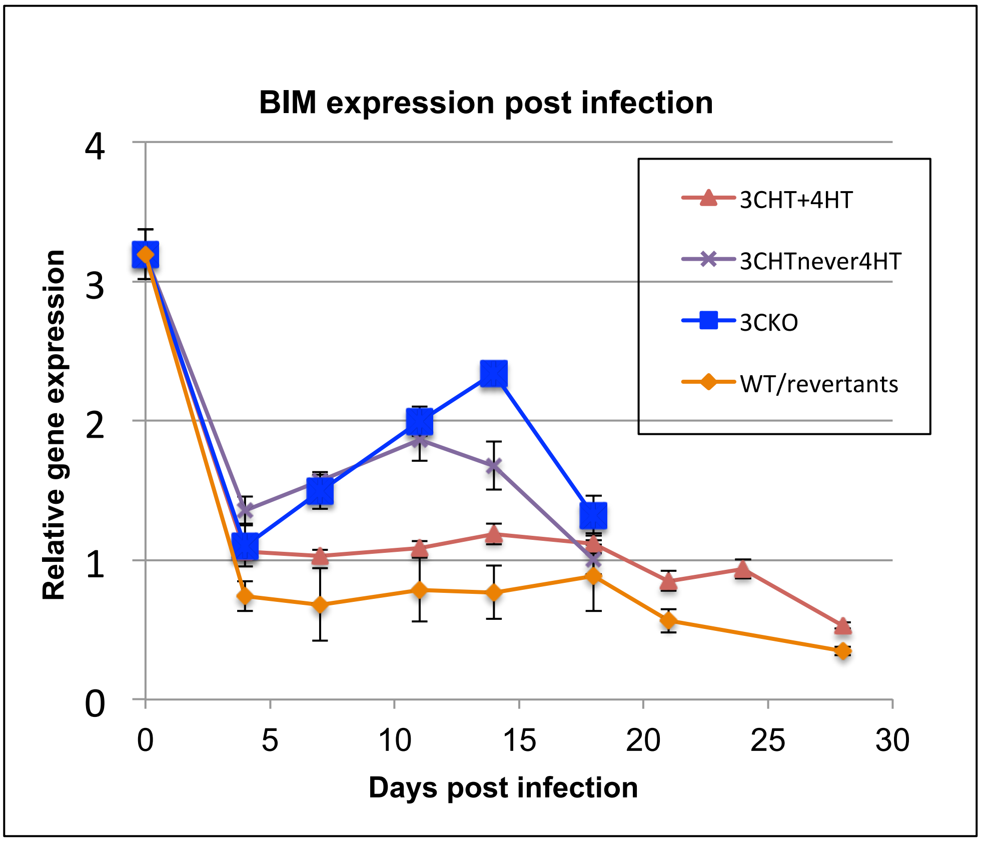

Figure S8. Changes in *BIM* (*BCL2L11*) gene expression after EBV infection of primary B cells. Expression of *BIM* RNA as measured by qPCR after infection with wild-type/revertant EBV-BACs, as compared to 3CKO EBV. The orange line represents the average expression (and standard deviation) of independent infections with four different wild-type or revertant EBVs. This is compared to infections with 3CKO (blue) and 3CHT in the absence of 4HT (purple) and with 3CHT+4HT (red), where the error bars indicate standard deviation of triplicate qPCRs. Expression data were normalized to expression of *ALAS1*, *GNB2L1* and *RPLP0* and expression values are expressed relative to the average of all data points. Note the higher *BIM* expression in the EBNA3C-deficient infections, in keeping with EBNA3C's role as a repressor of BIM transcription. Also, as seen for $p16^{INK4A}$ expression (Figure 7), there is a slightly reduced efficiency of repression of *BIM* by the 3CHT virus grown with 4HT as compared to wild-type viruses. The drop in *BIM* RNA levels after two weeks probably occur because cells with higher *BIM* RNA levels die as a critical threshold of BIM protein is passed.
